# Structural, optical, thermal, and computational insights into the 1D antimony-based hybrid perovskite (C_10_H_13_N_4_)[SbI_4_]·H_2_O

**DOI:** 10.1039/d6ra02020h

**Published:** 2026-05-05

**Authors:** Amin Alibi, Nour Elleuch, Sergiu Shova, Frédéric Amiard, Jerome Lhoste, Mohamed Boujelbene

**Affiliations:** a Laboratory of Physico-Chemistry of Solid State, LR11ES51, Sfax Faculty of Sciences, University of Sfax Sfax 3000 Tunisia m_boujelbene2010@yahoo.fr; b “Petru Poni” Institute of Macromolecular Chemistry Alea Grigore Ghica voda 41-A 700487 Iasi Romania; c MMM-UMR 6283 CNRS, Lunam, Faculty of Sciences and Techniques, University of Maine Avenue Olivier Messiaen 72085 Le Mans Cedex 9 France

## Abstract

A new one-dimensional organic–inorganic hybrid antimony halide, (C_10_H_13_N_4_)[SbI_4_]·H_2_O, was synthesized *via* a hydrothermal method and structurally characterized by single-crystal X-ray diffraction. The crystal structure comprises infinite [SbI_4_]^−^ chains separated by protonated organic cations and lattice water molecules, forming a supramolecular architecture stabilized by hydrogen bonding and other noncovalent interactions. Phase purity was confirmed by powder X-ray diffraction. At the same time, Hirshfeld surface and intermolecular energy analyses reveal that electrostatic interactions and hydrogen bonding play a dominant role in the crystal packing. Vibrational properties investigated by FT-IR and Raman spectroscopy, supported by density functional theory calculations, confirm the structural integrity of both the organic and inorganic components. Optical studies show visible photoluminescence under UV excitation, and the optical band gap is estimated to be about 3.06 eV, while theoretical calculations predict a band gap close to 2.9 eV, indicating semiconducting behavior. Thermal analysis further demonstrates a well-defined multistep decomposition process consistent with the structural composition. These results highlight the structural stability and optoelectronic potential of this new antimony-based hybrid material.

## Abstract 2

A new one-dimensional organic–inorganic hybrid antimony halide, (C_10_H_13_N_4_)[SbI_4_]·H_2_O, was synthesized *via* a hydrothermal method and structurally characterized by single-crystal X-ray diffraction.

## Graphical abstract

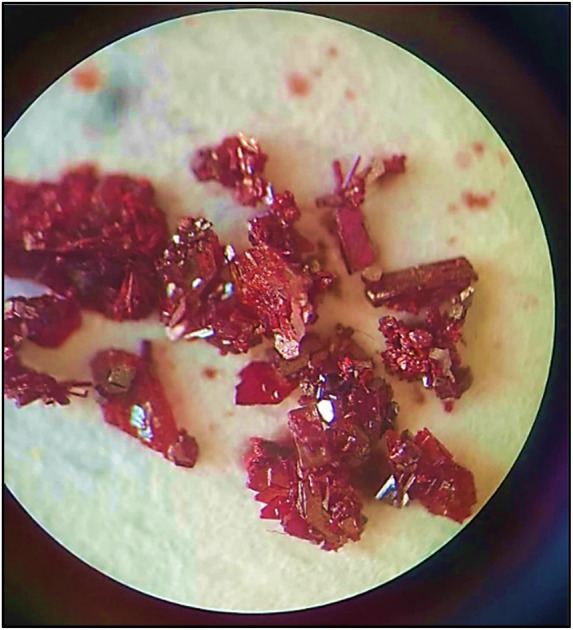

## 1. Introduction

Hybrid metal–halide perovskites have gained significant attention in recent years due to their remarkable structural versatility, tunable optoelectronic properties, and wide range of potential applications.^1,2^ These materials, particularly one-dimensional (1D) hybrid halide perovskites, have emerged as promising candidates for next-generation optoelectronic devices, including photovoltaics, light-emitting diodes, and photodetectors.^3,4^ The rational design of new hybrid perovskites with enhanced stability, lower energy gaps, and improved charge transport characteristics remains an area of intense research interest.^5^ In this work, we present a novel 1D hybrid metal–halide perovskite, (C_10_H_13_N_4_)[SbI_4_]·H_2_O, which demonstrates superior stability, a reduced energy gap, and a rich network of supramolecular interactions that contribute to its unique optical behavior.^6^

A key motive behind this study is the need to develop more stable and efficient hybrid perovskites by fine-tuning their structural and electronic properties.^7^ While three-dimensional (3D) perovskites have dominated the field due to their excellent charge transport and high absorption coefficients, their inherent instability under environmental conditions has driven interest toward lower-dimensional counterparts.^8^ Among these, 1D perovskites stand out due to their enhanced moisture resistance, reduced defect densities, and tunable optoelectronic properties.^9^ Our newly synthesized compound, (C_10_H_13_N_4_)[SbI_4_]·H_2_O, represents a strategic improvement over previously reported 1D BiBr_4_-based analogs, exhibiting a lower energy gap than 3.29 eV and a more intricate supramolecular framework, leading to stronger intermolecular interactions and enhanced stability. This enhancement is critical for expanding the application range of 1D perovskites in energy-related technologies.^10^

The introduction of antimony (Sb) in place of bismuth (Bi) is a deliberate choice aimed at further optimizing the electronic structure and stability of the perovskite framework.^11^ Antimony-based perovskites have demonstrated competitive bandgap tunability and strong spin–orbit coupling effects, making them viable alternatives to lead-based systems.^12^ Moreover, iodine (I) was selected as the halogen component due to its superior ability to extend light absorption into the visible range and reinforce the overall stability of the inorganic framework.^13^ This specific composition leads to a perovskite material with strong photoluminescence, making it a highly desirable candidate for optoelectronic applications.^14^

Despite the growing interest in hybrid halide perovskites, many knowledge gaps remain, particularly concerning the role of supramolecular interactions in governing their optical and electronic properties.^15^ Our study addresses these gaps by providing an in-depth structural analysis of (C_10_H_13_N_4_)[SbI_4_]·H_2_O, elucidating the contributions of halogen bonding, hydrogen bonding, and π–π interactions in shaping its physicochemical behavior.^16^ Furthermore, the photophysical properties of this material are systematically investigated to correlate its structural features with its optical performance.^17^ This approach provides fundamental insights into the interplay between crystal structure and electronic properties in low-dimensional perovskites, paving the way for future material optimization.^18^

In addition to fundamental interest, our work aligns with the broader goal of developing perovskite-based materials for real-world applications. The unique combination of enhanced stability, strong optical absorption, and a well-defined supramolecular framework positions this material as a potential candidate for optoelectronic technologies, including photodetectors, luminescent materials, and sensing applications. Furthermore, the tunable nature of hybrid perovskites suggests that similar materials could be designed to meet the demands of next-generation energy storage and conversion technologies.

Ultimately, this study contributes to the ongoing efforts to optimize hybrid perovskites by strategically modifying their composition and structure to achieve superior properties. By highlighting the advantages of 1D perovskites and elucidating the structure–property relationships in (C_10_H_13_N_4_)[SbI_4_]·H_2_O, we provide a comprehensive understanding of how these materials can be further developed for advanced optoelectronic applications. The insights gained from this work will serve as a valuable foundation for future research in the field of low-dimensional hybrid perovskites, ultimately advancing the quest for high-performance, stable, and environmentally friendly perovskite materials.^19^

## 2. Experimental

### 2.1. Materials and characterization techniques

Both SbI_3_ and HI, obtained commercially, were used as received alongside the non-commercial organic molecule (C_10_H_12_N_4_) to synthesize our novel 1D antimony-based hybrid material. The synthesis was conducted under controlled conditions, yielding a crystalline product that underwent comprehensive characterization. Single-crystal X-ray diffraction (SC-XRD) was employed to precisely determine the compound's atomic arrangement and structural integrity. Optical absorption properties were investigated using UV-Vis spectroscopy in both aqueous and solid states (DRS). In aqueous solution, spectra were acquired with a Cary 5000 UV-Vis-NIR spectrophotometer (250–600 nm range), while solid-state spectra were obtained with a PerkinElmer Lambda 35 UV-Vis spectrophotometer equipped with an integrating sphere (200–1100 nm range). Photoluminescence (PL) spectroscopy provided insights into its emission characteristics at room temperature. Vibrational features were analyzed by Fourier-transform infrared (FTIR) spectroscopy (PerkinElmer FTIR Spectrometer, 4000–500 cm^−1^) and Raman spectroscopy (Horiba/Jobin Yvon T6400, 4000–50 cm^−1^). Thermal stability was assessed through thermogravimetric analysis (TGA) and differential scanning calorimetry (DSC) under controlled heating conditions. TGA was carried out using a multi-module 92 SETA RAM analyzer at a constant rate of 10 °C min^−1^ from room temperature up to 600 °C under N_2_ inert gas, while DSC was carried out using a PerkinElmer DSC 4000 calorimeter, also in an inert nitrogen atmosphere (N_2_), with a heating and cooling rate of 5 °C min^−1^ over a temperature range from 25 °C to 350 °C. Additionally, Density Functional Theory (DFT) computational studies were conducted to analyze the compound's electronic structure and charge distribution, offering theoretical insights into its optoelectronic behavior.

### 2.2. Synthesis of (C_10_H_13_N_4_)[SbI_4_]·H_2_O

**Fig. 1:**
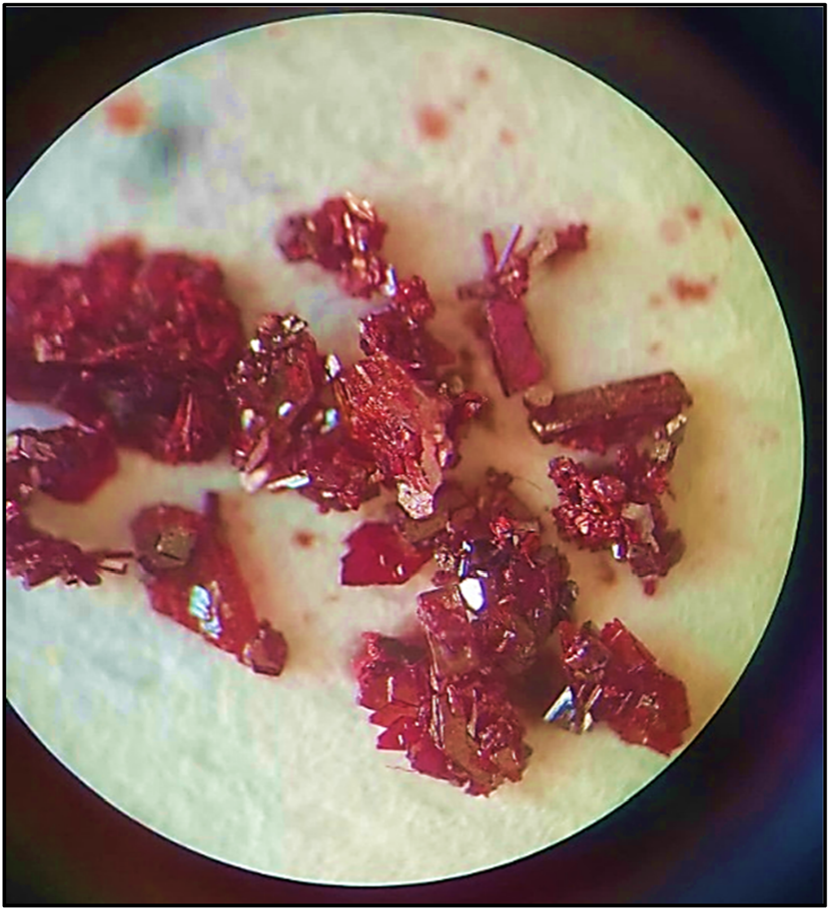
Macroscopic view of the single crystals of the hybrid material.

Single crystals of the hybrid compound (C_10_H_13_N_4_)[SbI_4_]·H_2_O were obtained using a hydrothermal synthesis approach. The organic precursor 5-amino-3-ethyl-1-phenyl-1*H*-1,2,4-triazole (C_10_H_12_N_4_), previously synthesized in our laboratory and not commercially available, was dissolved in distilled water together with SbI_3_ (98%) in an appropriate stoichiometric proportion. Subsequently, a few drops of concentrated hydroiodic acid (HI, 57%) were added to the solution in order to ensure complete dissolution of the inorganic precursor and to provide the iodide-rich acidic medium required for the formation of the tetraiodoantimonate anion. The resulting homogeneous mixture was transferred into a Teflon-lined stainless-steel autoclave, sealed, and heated at 140 °C for four days under autogenous pressure. After slow cooling to room temperature, well-formed crystals suitable for single-crystal X-ray diffraction analysis were isolated from the mother liquor and carefully selected for structural characterization (Fig. 1).

Complementary structural characterization was performed by powder X-ray diffraction (PXRD), presented in of Fig. 1.S. The experimental diffraction pattern (blue curve) exhibits sharp and well-defined reflections, confirming the high crystallinity of the sample, and shows excellent agreement with the simulated pattern calculated from the single-crystal structural data (black curve). The close correspondence in both peak positions and relative intensities indicates the absence of secondary phases and confirms the validity of the structural model proposed for the hybrid antimony–iodide compound (C_10_H_13_N_4_)[SbI_4_]·H_2_O. These PXRD results therefore verify the phase purity and structural consistency of the synthesized material.

### 2.3. Single-crystal X-ray data collection and structure determination

The crystal structure of (C_10_H_13_N_4_)[SbI_4_]·H_2_O was precisely determined *via* single-crystal X-ray diffraction (SCXRD) at 293 K. Data reduction and structural analysis were performed using the Olex2-1.5 software package,^20^ with the initial structure solution obtained through direct methods (SHELXT 2018/2 (ref. 21)) and refined using full-matrix least-squares refinement on F^2^ (SHELXL 2018/3 (ref. 22)). The experiment employed Mo-Kα radiation (*λ* = 0.71073 Å) on an XtaLAB Synergy diffractometer equipped with a Dualflex source and a HyPix detector, utilizing a carefully selected crystal with dimensions of 0.15 × 0.15 × 0.04 mm^3^. Anisotropic displacement parameters were assigned to all non-hydrogen atoms, while hydrogen atoms were positioned using the HFIX command under specific constraints: CH_2_ and NH_2_ were set to 23, CH_3_ to 137, NH and aromatic CH to 43, and H_2_O to 6. The refinement converged to outstanding reliability factors (*R*_1_ = 0.053, w*R*_2_ = 0.084), reflecting the high precision of the structural model. To illustrate the molecular organization, intermolecular interactions, and crystallographic symmetry, DIAMOND 3 software^23^ was utilized for generating structural representations and packing diagrams. Comprehensive crystallographic refinement details, including key structural parameters, are systematically compiled in Table 1.

**Table 1:** Summary of crystal data and structure refinement details

Crystallographic data	
Empirical formula	**(C_10_H_13_N_4_)[SbI_4_]·H_2_O**
Shape/color	Prism, dark deep red
Molar mass (g mol^−1^)	836.61
Diffractometer	XtaLAB Synergy, Dualflex, HyPix
Radiation type	*λ*(Kα) = 0.71073 Å (Mo Kα radiation)
Temperature (K)	293
Calculated density (mg m^−3^)	2.732
Crystal system	Monoclinic
Space group	*P*2_1_/*n*
*Z*/*Z*′	4/1
Unit cell parameters	
*a* (Å)	10.3400 (5)
*b* (Å)	7.7127 (3)
*c* (Å)	25.5169 (10)
*β* (°)	91.456 (4)
Absorption coefficient (mm^−1^)	7.43
Number of reflections measured variation of *h*, *k*, *l*	*h* = −12–12, *k* = −9–9, *l* = −30–29
Scanning range of *θ* (°)	2.1–25
*F*(000)	1496
Independent parameters	187
Δ*ρ*_max_/Δ*ρ*_min_ (e Å^−3^)	0.81/−0.61
(Δ/*σ*)_max_	0.001
*R*[*F*^2^ > 2*σ*(*F*^2^)] = *R*_1_	0.053
w*R*(*F*^2^) = w*R*_2_	0.084
*S* = GooF	1.14
CCDC	**2431918**

### 2.4. Hirshfeld surface analysis

Hirshfeld surface analysis^24–27^ was carried out using CrystalExplorer to evaluate the intermolecular interactions governing the crystal packing of the synthesized compound. The structural input was obtained from single-crystal X-ray diffraction data, ensuring accuracy in the electron density calculations. The analysis involved generating Hirshfeld surfaces mapped with dnorm, shape index, and curvedness properties to visualize and interpret non-covalent interactions in three-dimensional space.

2D Fingerprint plots^28,29^ were constructed to quantify the relative contributions of different interaction types. These plots were generated by analyzing pairwise contacts, allowing for a detailed examination of hydrogen bonding, halogen···halogen interactions, π⋯π stacking, and other van der Waals forces.^30,31^ The software computed the percentage contributions of each type of interaction, facilitating a comparative assessment of their influence on the crystal structure.

Additionally, dnorm surface mapping was employed to highlight close-contact regions, where interactions shorter than the sum of van der Waals radii appear as red regions, while longer or non-interacting regions appear in white and blue, respectively. The shape index and curvedness plots provided further insight into the molecular surface topology, revealing the extent of planar stacking and π⋯π interactions.

All calculations and visualizations were performed within CrystalExplorer 21.5,^32^ using standard computational parameters to ensure the reliability of the generated Hirshfeld surfaces and fingerprint plots.

### 2.5. Computational details

The computational study of the hybrid material (C_10_H_13_N_4_)[SbI_4_]·H_2_O was conducted to elucidate its structural stability, electronic characteristics, and optical behavior. All calculations were performed within the framework of density functional theory (DFT), employing the B3LYP functional^33^ and the LanL2DZ basis set,^34^ as implemented in the Gaussian 09W program.^35^ To accurately model intermolecular interactions and their influence on the system's properties, a representative cluster was constructed, comprising a single water molecule (H_2_O), an organic cation (C_10_H_13_N_4_)^+^, and an anionic [SbI_4_]^−^ unit. The entire system was fully optimized, allowing all atomic positions to relax, ensuring that the final structure corresponded to an energy-minimized state.

To further analyze the vibrational properties, frequency calculations were carried out, enabling direct comparison with experimental spectra. Mode assignments were derived primarily from DFT results, with vibrational motion visualized using GaussView 6.0.16.^36^ Electronic structure investigations included the computation of the partial density of states (PDOS), electron localization function (ELF), localized orbital locator (LOL), and electrostatic potential (ESP), all performed using Multiwfn software^37^ to capture key bonding characteristics and charge distribution trends. Additionally, simulated UV-Vis absorption spectra were obtained and systematically compared with experimental data, providing deeper insight into the material's optical properties.

The decision to forgo the GENECP option stemmed from two key considerations. First, its significantly higher computational cost and time requirements made it less practical than the LANL2DZ basis set, which offered a more efficient approach without substantial loss of accuracy. Second, while GENECP is widely recognized for its precision in describing heavy metal elements, benchmarking revealed that LANL2DZ produced results with only minimal deviations from GENECP-derived values. Given the strong agreement between both methods, the use of the more computationally demanding GENECP was deemed unnecessary for this study, ensuring an optimal balance between accuracy and efficiency.

## 3. Results and discussion

### 3.1. Crystal structure

**Fig. 2:**
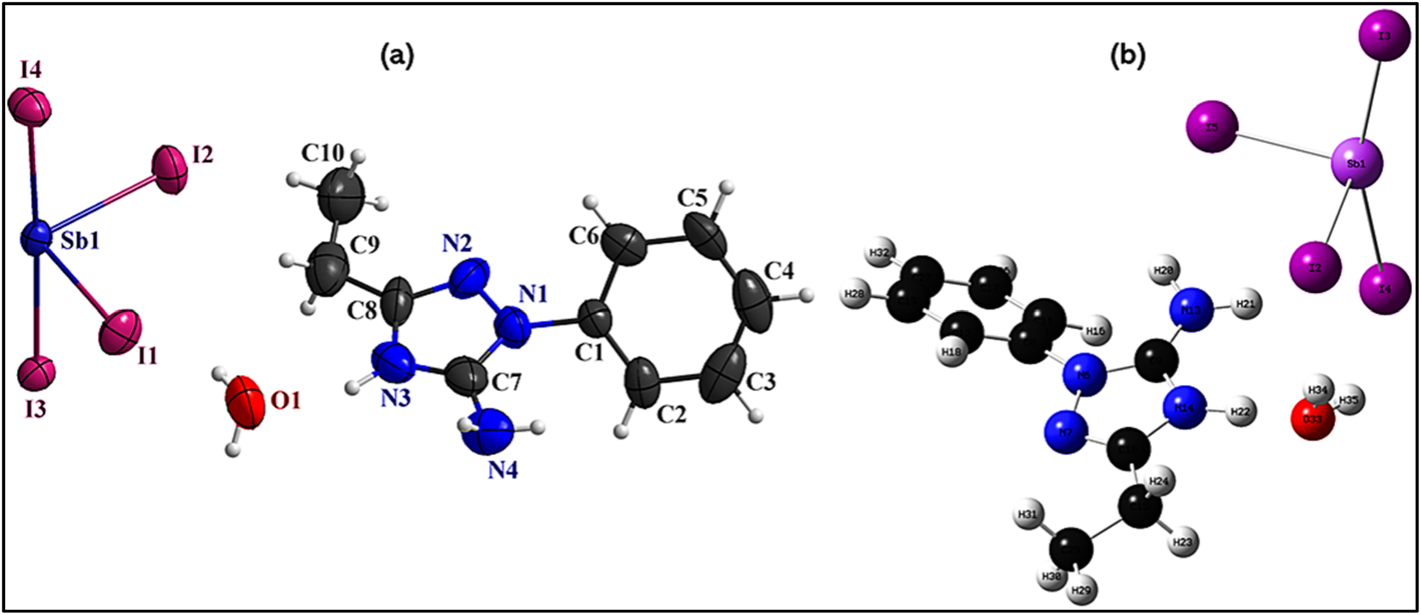
(a) Experimental, and (b) theoretical formula units.

The asymmetric unit of the 1D hybrid organic–inorganic compound (C_10_H_13_N_4_)[SbI_4_]·H_2_O (Fig. 2) comprises a protonated organic cation, an anionic [SbI_4_]^*n*−^ chain, and a crystallized water molecule that collectively governs the structural organization and stability. The compound crystallizes in the monoclinic space group *P*2_1_/*n* with unit-cell parameters *a* = 10.3400(5) Å, *b* = 7.7127(3) Å, *c* = 25.5169(10) Å, and *β* = 91.456(4)°, giving a volume of 2034.30(15) Å^3^ and a calculated density of 2.732 mg m^−3^.

**Fig. 3:**
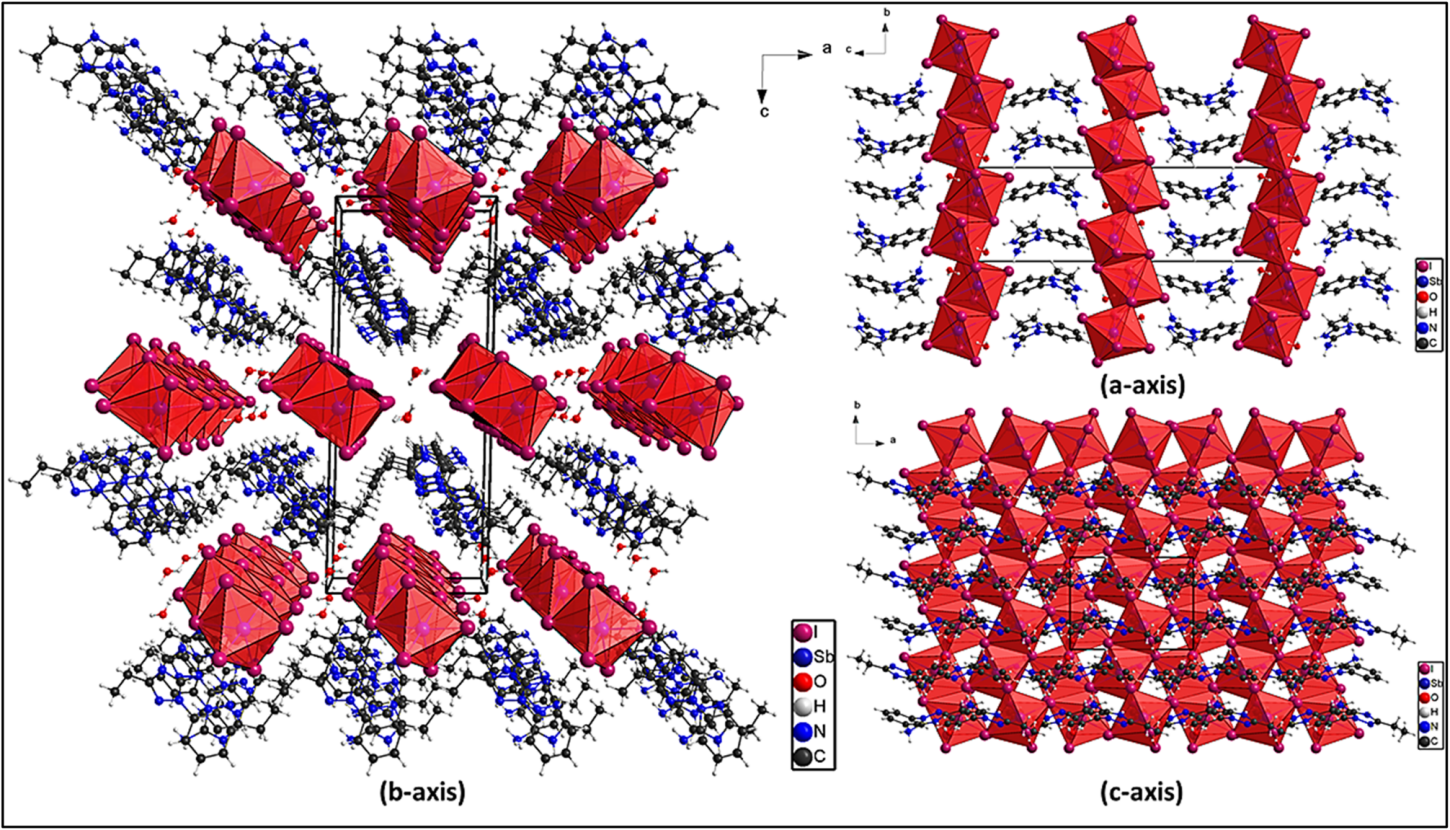
Projections of the unit cell along the three axes.

**Fig. 4:**
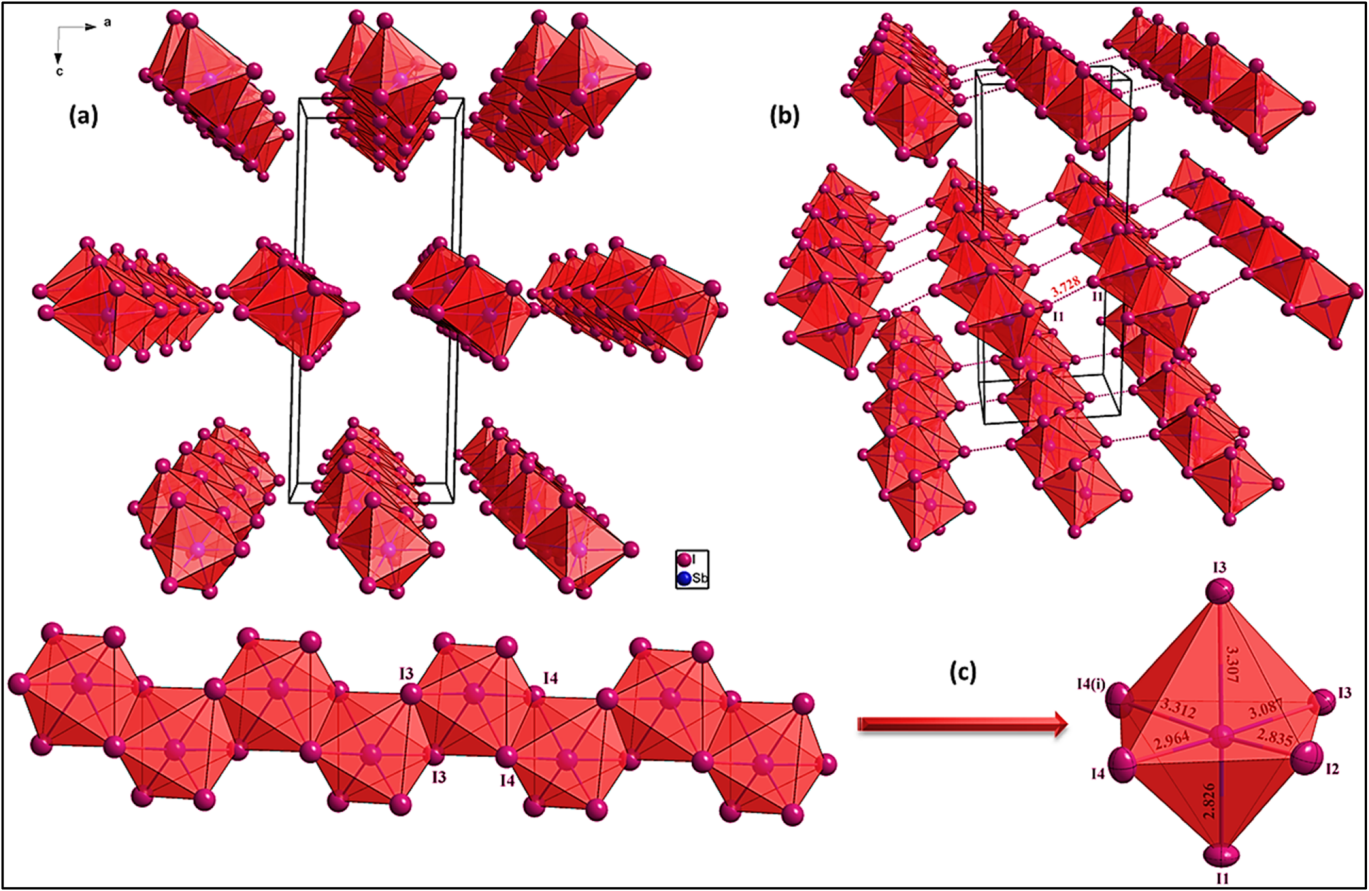
(a) Projection of the anionic chains along the *b*-axis, (b) halogen–halogen (I⋯I) interactions, and (c) the distortion of the polyhedron.

The inorganic [SbI_4_]^*n*−^ framework exhibits a seesaw coordination geometry around Sb(iii), resulting from the stereochemically active lone pair that distorts the coordination environment from ideal tetrahedral symmetry. The four iodine ligands bind asymmetrically, producing distinct equatorial and axial Sb–I distances (discussed in Fig. 4). Bridging Sb–I interactions propagate the structure into a continuous one-dimensional chain along a crystallographic direction, defining the structural dimensionality and contributing to lattice stability and electronic behavior. The protonated (C_10_H_13_N_4_)^+^ cation ensures charge balance and stabilizes the lattice through hydrogen-bond interactions with the water molecule and surrounding iodide atoms (Fig. 3.S). These interactions regulate the spacing between neighboring chains and prevent excessive aggregation, preserving an ordered packing arrangement and influencing intermolecular interactions within the crystal.

The experimental (Fig. 2(a)) and theoretical (Fig. 2(b)) representations of (C_10_H_13_N_4_)[SbI_4_]·H_2_O show strong agreement, with only minor deviations arising from the finite molecular model used in calculations *versus* the periodic crystalline environment. The most noticeable difference concerns the axial I–Sb–I angle and related bond distances, which vary slightly in the theoretical model because crystal packing and non-covalent interactions are absent. Small variations also appear within the organic cation geometry but remain negligible, confirming the adequacy of the computational approach and basis sets. Furthermore, the crystallized water molecule present in the experimental lattice contributes to supramolecular stabilization through hydrogen bonding with nearby iodide ions and cations, an effect not included in the isolated theoretical model and therefore responsible for the remaining minor structural discrepancies.

The overall agreement between theoretical and experimental data supports the accuracy of the computational model, confirming that the theoretical approach provides a reliable representation of the electronic structure and molecular geometry, despite the inherent simplifications of gas-phase calculations. The small discrepancies in bond angles and distances highlight the importance of including periodic interactions when modeling hybrid systems, especially for understanding packing effects, hydrogen bonding, and crystal field influences.

The structural projections along the *a*-, *b*-, and *c*-axes shown in Fig. 3 provide a clear visualization of the molecular arrangement and packing architecture within the 1D antimony-based hybrid organic–inorganic framework. The [SbI_4_]^*n*−^ anionic chains extend along a crystallographic direction, forming a well-defined 1D coordination network through Sb–I bridging interactions. The inversion center represents the key symmetry element controlling the connectivity, determining the spatial relationships between neighboring [SbI_4_]^*n*−^ chains, organic cations, and crystallized water molecules. The organic cations and water molecules occupy interstitial sites, maintaining electrostatic balance and stabilizing the extended framework through hydrogen-bond interactions.

Viewed along the *a*-axis, the projection emphasizes the 1D nature of the [SbI_4_]^*n*−^ anionic chains, which propagate through Sb–I bridges to form a polymeric structure rather than isolated 0D anions. These chains are separated by the organic cations that act as charge-compensating species. Structural periodicity is reinforced by hydrogen bonds involving the cations and the lattice water molecule (discussed in Fig. 3.S). The absence of direct Sb–Sb interactions preserves the integrity of the 1D coordination framework and maintains the localized electronic environment around each Sb(iii) center.

The projection along the *b*-axis highlights the arrangement of organic cations relative to the anionic chains. The cations are organized to favor π–π stacking interactions, generating alternating organic subchains with intermolecular distances of 3.839 Å and 3.888 Å that strengthen the supramolecular packing (further examined in Fig. 4). Crystallized water molecules occupy interstitial positions and participate in hydrogen bonding that links adjacent components, reinforcing lattice stability.

Along the *c*-axis, the periodic nature of the lattice becomes evident, revealing an ordered assembly of anionic chains and organic cations. Inversion symmetry produces a regular distribution of [SbI_4_]^*n*−^ chains within the unit cell. The organic cations function as spacers, maintaining separation between inorganic chains while noncovalent interactions collectively contribute to a stable and well-organized crystal structure.

The anionic entity [SbI_4_]^−^, as part of the extended 1D chain, exhibits a distorted octahedral coordination around the Sb(iii) center, influenced by the stereochemically active lone pair of antimony, as shown in Fig. 4(a). A detailed analysis of the Sb–I bond lengths, shown in Fig. 4(c), reveals two distinct sets of distances, with the equatorial bonds being notably shorter than the axial bonds, a typical feature of lone-pair-influenced geometries. The degree of distortion from an idealized octahedral geometry is quantified by the geometric distortion index (ID):
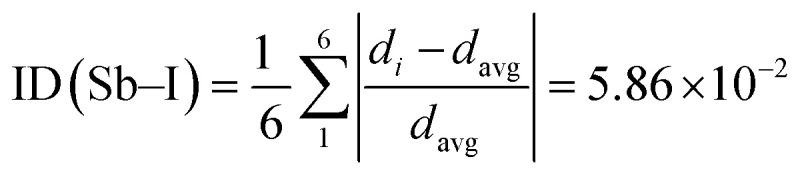
where the variables *d*_*i*_ (*i* = 1 to 6) represent the individual Sb–I bond lengths, and *d*_avg_ denotes the mean bond length within the distorted octahedral unit. This calculation offers a quantitative measure of structural deviation, facilitating a direct evaluation of distortions caused by packing constraints, halogen interactions, or electronic effects. By comparing the individual bond lengths to the average, this method provides insight into the degree of distortion within the distorted octahedron structure, enhancing our understanding of the factors influencing bond geometry. The calculated distortion index (5.86%) confirms that the octahedral coordination significantly deviates from an ideal geometry, with the axial Sb–I bonds being substantially elongated compared to their equatorial counterparts.

This distortion directly influences the electronic properties of the material by affecting orbital hybridization and charge distribution around the Sb(iii) center. The asymmetry in bond lengths leads to localized electronic anisotropy, which impacts optical absorption behavior, which is a characteristic commonly observed in 1D heavy poor metal–halide hybrid materials. Furthermore, the halogen–halogen interactions play a crucial role in stabilizing the 1D chain, reinforcing the solid-state packing, and potentially modulating the optoelectronic properties of the material.

The halogen bonding network, presented in Fig. 4(b), plays a crucial role in stabilizing the solid-state packing of the material. The presence of short I⋯I interactions between adjacent [SbI_4_]_*n*_^−^ chains suggest the formation of a supramolecular halogen bonding framework that significantly contributes to lattice cohesion. A comparative analysis of these I⋯I distances with known literature values for halogen bonding places them firmly within the range of type-II halogen bonding, which typically occurs between 3.50 and 3.90 Å. These interactions confirm their noncovalent stabilizing role, where the anisotropic electron distribution around iodine atoms leads to attractive electrostatic interactions, further reinforcing the 1D coordination network rather than an isolated 0D assembly.

The nature and significance of these halogen bonds are reinforced by:

• Directional preference, where the contacts align with the σ-hole regions of the iodine atoms, enhancing their electrostatic attraction.

• Packing effects, which optimize these interactions without leading to steric hindrance.

Such interactions are known to influence optoelectronic properties, particularly by modifying the band gap and influencing charge carrier mobility, making this an important structural feature to consider in the functional context of the material. The polyhedral distortion within the [SbI_4_]_*n*_^−^ chain, combined with halogen bonding interactions, collectively contributes to the electronic structure modulation of the material. The elongation of axial Sb–I bonds, coupled with the anisotropic electron density distribution, suggests a potential impact on:

• Optical absorption behavior, as distortions in the coordination environment influence the frontier molecular orbitals.

• Charge localization and transport, where deviations from idealized bonding geometries may affect excitonic properties.

Future computational studies could provide deeper insight into how these structural factors contribute to electronic transitions, particularly in relation to potential semiconducting or optoelectronic applications.

The structural projection along the *a*-axis (Fig. 2.S(a)) reveals a complex network of π-based interactions, where two π⋯π stacking, two N–H⋯π, two C–H⋯π, and two n⋯π interactions alternate systematically along the *b*-axis, generating 1D chains of organic cations. These interactions play an important role in stabilizing the hybrid framework and strongly influence the supramolecular organization and packing efficiency of the structure.

Within the unit cell, these interactions create two distinct 1D chains composed of stacked organic cations oriented along the *b*-axis. The stacking pattern follows a clear periodicity defined by two alternating π–π interaction distances of 3.839 Å and 3.888 Å. These contacts arise from face-to-face interactions between aromatic rings, promoting molecular self-assembly and partial electronic delocalization. The combined effect of π–π stacking with other noncovalent forces, including hydrogen bonding (Fig. 3.S) and halogen interactions (Fig. 4), contributes to a more complex three-dimensional packing arrangement that enhances lattice stability.

The π–π stacking originates from interactions between conjugated aromatic rings of the organic cation. The alternating distances between adjacent rings indicate a parallel-displaced stacking mode, which is energetically more favorable than perfect face-centered stacking because it minimizes electrostatic repulsion and steric hindrance. Analysis of centroid-to-centroid distances and slippage values confirms the presence of offset π-stacking, a characteristic feature of supramolecular interactions in hybrid materials.

The centroid-to-centroid separations (Cg⋯Cg) of 3.888 and 3.823 Å fall within the typical range for effective π–π stacking (3.3–4.0 Å), confirming moderate to strong attractive interactions between neighboring aromatic rings. The existence of two distinct distances indicates alternating stacking interactions rather than a uniform arrangement, likely caused by subtle variations in molecular orientation or neighboring intermolecular forces.

Although the exact interplanar distance is unavailable, the non-zero slippage angle of 3.246° indicates a parallel-displaced configuration rather than perfectly eclipsed stacking. In such geometries, the interplanar separation is generally slightly shorter than the centroid distance, allowing favorable orbital overlap while limiting electrostatic repulsion between adjacent π-electron systems.

Beyond π⋯π stacking (Fig. 2.S(b)), N–H⋯π (Fig. 2.S(b)), C–H⋯π (Fig. 2.S(c)), and n⋯π (Fig. 2.S(d)) interactions contribute significantly to the long-range organization and structural locking of the organic cations within the lattice:

• N–H⋯π interactions (2.854 and 3.676 Å) arise from the direct interaction of the protonated nitrogen donors of the organic cations with the π-cloud of adjacent aromatic rings, creating a directional force that further stabilizes the π-stacked framework.

• C–H⋯π interactions (3.623 (C3–H3⋯π) and 3.760 Å (C5–H5⋯π)) reinforce non-classical hydrogen bonding, as hydrogen atoms from aliphatic and aromatic groups engage in π-orbital interactions, enhancing lattice cohesion. These interactions fine-tune the interlayer organization without introducing significant steric effects.

• n⋯π interactions (3.464 (N4) and 3.538 Å (N2)) result from the lone pairs of heteroatoms engaging with adjacent π-systems, effectively modulating charge distribution and stabilizing π-electron delocalization.

Together, these interactions define a hierarchical supramolecular assembly, where π-related forces alternate, ensuring uniform packing and minimizing steric distortions.

When evaluating the relative significance of these π interactions in this structure, it is essential to compare their strength and role against other key noncovalent interactions, particularly halogen–halogen (I⋯I) bonding and hydrogen bonding (discussed in Fig. 3.S). After comparing these non-covalent interactions, a clear structural hierarchy emerges:

• Halogen–halogen (I⋯I) interactions (Fig. 4) are electrostatic, directional noncovalent contacts that significantly stabilize the inorganic framework by reinforcing the packing order of the [SbI_4_]_*n*_^−^ anionic chains. These I⋯I interactions control the spatial arrangement of the anions, preserving structural stability while maintaining the 1D polymeric connectivity of the [SbI_4_]_*n*_^−^ chains. In contrast, π–π interactions act as secondary stabilization forces operating mainly within the organic sublattice without directly affecting the Sb–I framework.

• Hydrogen bonding interactions (Fig. 3.S), particularly N–H⋯O contacts, are highly directional and charge-assisted, producing stronger stabilization per interaction than π stacking. However, π-related interactions (π⋯π, N–H⋯π, C–H⋯π, and n⋯π) collectively provide long-range stabilization, governing the organic sublattice organization and promoting molecular alignment and electronic delocalization without significantly altering the geometry of the inorganic units.

As a conclusion, Hydrogen bonding is stronger and more directional, while π stacking contributes long-range stabilization and planar assembly. In this structure, hydrogen bonds serve as the primary stabilizing force, whereas π stacking supplements structural integrity. Halogen bonding (X⋯X interactions) primarily influences anionic framework arrangement, whereas π-stacking impacts the cationic system. The interplay of halogen bonding and π stacking can modulate electronic structure, potentially affecting optical bandgap and electronic conductivity.

This comparative analysis shows that π interactions provide an additional stabilization mechanism that complements the primary interactions, namely I⋯I halogen bonding and hydrogen bonding. The combined presence of π⋯π stacking, N–H⋯π, C–H⋯π, and n⋯π interactions generates an extended 1D supramolecular chain along the *b*-axis, reinforcing the organic sublattice while cooperating with hydrogen and halogen bonding. Although individually weaker than hydrogen bonds, the cumulative influence of π interactions on charge distribution, packing efficiency, and molecular orientation makes them an important secondary stabilizing force.

While π stacking enhances molecular cohesion, it operates in balance with other noncovalent interactions. The interplay among these forces determines the supramolecular organization, electronic behavior, and overall stability of the crystal. Hydrogen and halogen bonding can strengthen or partially limit π stacking by modifying molecular alignment and packing density. In particular, the strong N–H⋯O hydrogen bonds (Fig. 3.S) may locally restrict molecular flexibility, preventing excessive distortions in π stacking and preserving electronic coherence within the aromatic systems.

The presence of π-stacking and alternating π-related interactions extends beyond mere structural stabilization, potentially influencing key material properties:

• Electronic structure properties: The overlapping π-electron clouds could modulate charge distribution and contribute to potential charge transport pathways. While the 1D nature of the inorganic [SbI_4_]_*n*_^−^ chain suggests a degree of electronic anisotropy, π-stacking interactions within the organic sublattice may still influence localized charge transfer and dielectric properties.

• Optical absorption: The periodicity of π-stacking distances could influence electronic delocalization, thereby modulating light absorption properties and charge carrier dynamics, particularly by affecting frontier molecular orbitals.

• Structural rigidity: The alternating nature of π-stacking interactions minimizes dynamic disorder, reinforcing mechanical stability and reducing lattice flexibility, which is essential for maintaining the integrity of the 1D framework.

• Thermal stability: The extended π-network may act as a thermal buffer, mitigating structural degradation under temperature fluctuations and enhancing the material's endurance, particularly by stabilizing both the organic cations and the [SbI_4_]_*n*_^−^ chain against thermal expansion effects.

Further spectroscopic and computational investigations could clarify the contribution of these π interactions to the functional properties of the material. In addition, the crystal framework contains hydrogen-bond interactions that significantly contribute to supramolecular organization (Fig. 3.S). Table 2 summarizes the geometric parameters of these interactions, including donor and acceptor atoms, hydrogen-bond distances (H⋯A and D⋯A), bond angles (D–H⋯A), and the symmetry operations generating the interactions.

**Table 2:** Interatomic distances and hydrogen bond angles of the compound^a^

D–H···A	D–H (Å)	H⋯A (Å)	D⋯A (Å)	D–H···A (°)
O1–H1B⋯O1^i^	0.85	2.85	3.345 (18)	119
N3–H3A⋯O1	0.86	1.93	2.745 (12)	157

a Symmetry code: (i) −*x* + 1, −*y* + 1, −*z* + 1.

The hydrogen-bonding network in the structure involves two distinct donor–acceptor pairs:

• O1–H1B⋯O1^i^ (symmetry-generated), with an H⋯A distance of 2.85 Å, donor–acceptor distance (D⋯A) of 3.345 Å, and bond angle (D–H⋯A) of 119°, corresponds to a moderately strong O–H⋯O hydrogen bond. This interaction likely involves the lattice water molecule (O1) acting simultaneously as donor and acceptor, generating intermolecular links within the supramolecular network. The relatively bent geometry (119°) suggests a somewhat reduced strength compared to linear hydrogen bonds. Nevertheless, this interaction contributes to lattice stabilization by connecting neighboring molecular units through oxygen-mediated hydrogen bonding and influencing the packing density of the structure.

• N3–H3A⋯O1 (intra–unit cell interaction) exhibits an H⋯A distance of 1.93 Å, a donor–acceptor distance (D⋯A) of 2.745 Å, and a bond angle of 157°. These parameters indicate a strong N–H⋯O hydrogen bond characterized by a short contact distance and a nearly linear geometry. The nitrogen donor (N3), belonging to the organic cation, participates in stabilizing the cationic network and enhancing molecular recognition within the crystal lattice. The short donor–acceptor separation reflects a strong electrostatic interaction that significantly reinforces the local packing environment.

The N–H⋯O interaction is stronger due to its shorter distance and more linear geometry, which enhances electrostatic attraction between the cationic and anionic components. In contrast, the O–H⋯O interaction, although weaker, acts as a structural bridge linking molecular units through water-mediated contacts. Together, these hydrogen bonds cooperate with π–π stacking interactions (Fig. 2.S) and halogen bonding (Fig. 4) to stabilize the 1D hybrid lattice.

Hydrogen bonds are highly directional and charge-driven, providing strong localized stabilization, whereas π stacking mainly contributes to electronic delocalization. Halogen bonding (I⋯I interactions) primarily stabilizes the anionic framework, while hydrogen bonding governs the cation–anion connectivity. The short and nearly linear N–H⋯O interaction enhances structural rigidity and may reduce molecular motion within the lattice.

Overall, the hydrogen-bond network strengthens crystal packing and complements π stacking and halogen interactions. While N–H⋯O bonds act as the dominant stabilizing force, O–H⋯O contacts function as secondary bridges that reinforce the supramolecular cohesion of the hybrid structure.

### 3.2. Vibrational studies

**Fig. 5:**
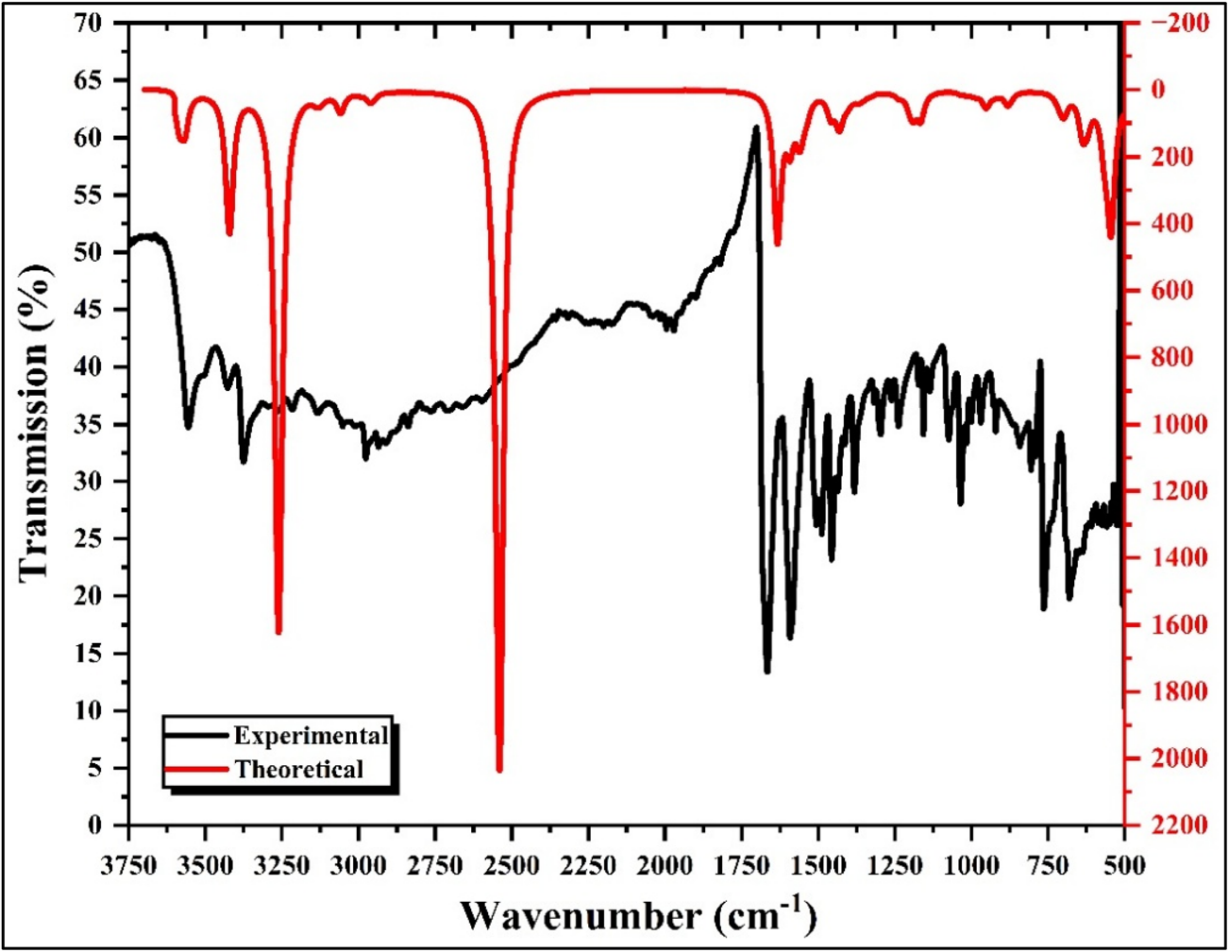
FT-IR spectrum of the title compound.

**Fig. 6:**
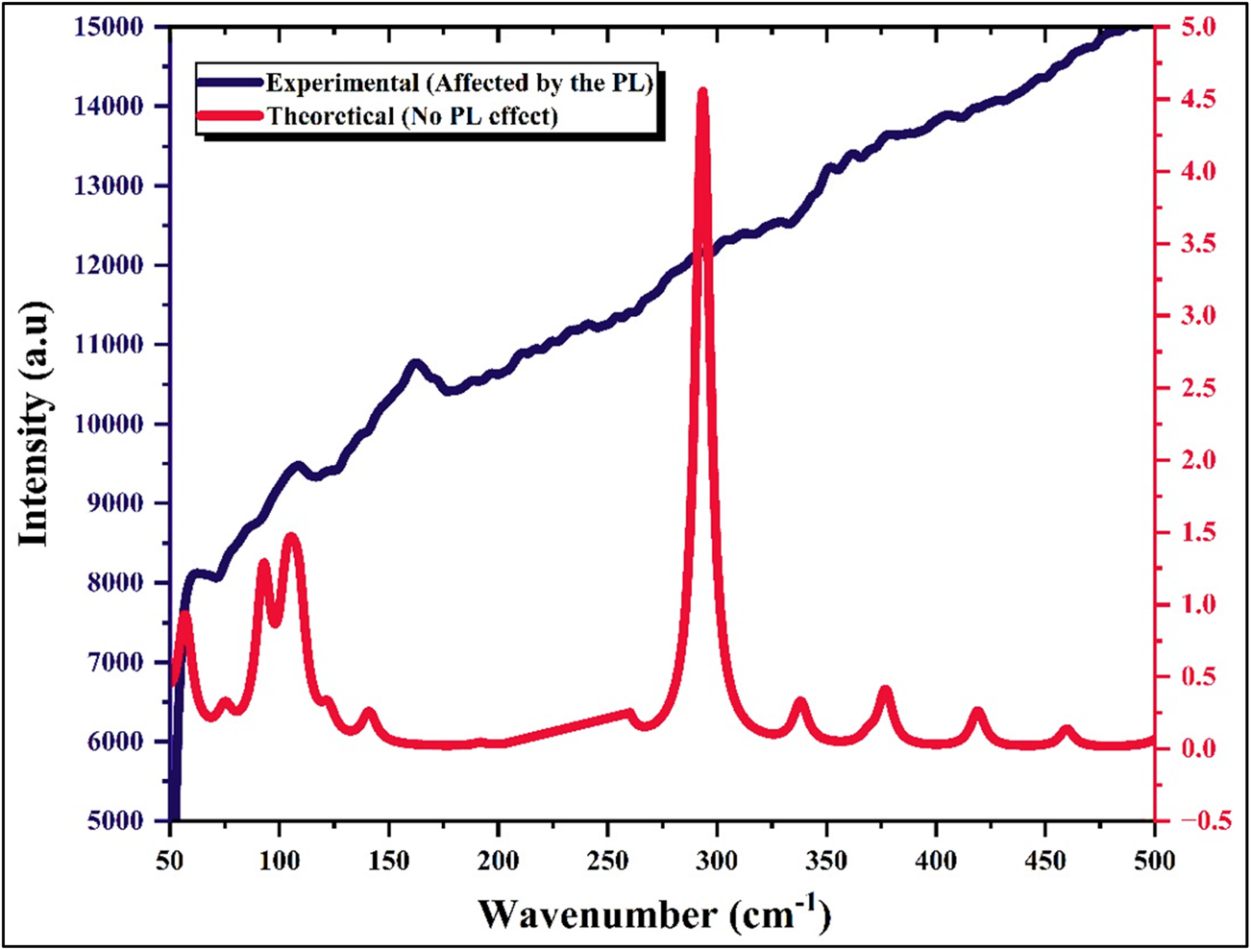
Raman spectrum of the title compound.

Vibrational spectroscopy was employed to investigate the molecular structure and lattice dynamics of the hybrid compound (C_10_H_13_N_4_)[SbI_4_]·H_2_O. The FT-IR and Raman spectra presented in Fig. 5 and 6, respectively, together with the calculated frequencies obtained from DFT and the corresponding assignments summarized in Table 1.S, provide a comprehensive description of the vibrational behavior of both the organic cation and the inorganic tetraiodoantimonate framework. The theoretical vibrational frequencies were obtained from harmonic calculations performed on the optimized structure and served as a reference for assigning the experimental bands. Minor deviations between experimental and calculated values originate from the harmonic approximation and the gas-phase conditions used in the calculations, whereas the experimental spectra reflect solid-state effects such as hydrogen bonding, intermolecular interactions, and crystal packing constraints.

#### 3.2.1. (C_10_H_13_N_4_)^+^ vibrational modes

The FT-IR spectrum is mainly dominated by the vibrational modes associated with the organic cation (C_10_H_13_N_4_)^+^ and the lattice water molecule. In the high-frequency region, the strong absorption band observed at 3556 cm^−1^ corresponds to the asymmetric stretching vibration of the NH_2_ group, while the band at 3362 cm^−1^ is attributed to its symmetric stretching mode. The presence of the lattice water molecule is confirmed by the band at 3427 cm^−1^, assigned to the symmetric stretching vibration of H_2_O, in good agreement with the calculated value. The aromatic and aliphatic C–H stretching vibrations appear in the 3129–2965 cm^−1^ region, where the band at 3129 cm^−1^ is associated with asymmetric C–H stretching of the aromatic ring, whereas the bands at 3052 cm^−1^ and 2965 cm^−1^ arise from combined symmetric C–H stretching and CH_3_/CH_2_ stretching modes of the ethyl substituent. A broad feature extending in the 1800–2800 cm^−1^ region is attributed to hydrogen-bonded *ν*(N–H⋯O) interactions, reflecting the involvement of the amino groups in intermolecular hydrogen bonding with the lattice water molecules, which contributes significantly to the stabilization of the hybrid crystal structure.

In the mid-frequency region, the band at 1668 cm^−1^ corresponds to a coupled vibration involving the H–O–H bending mode of water and the NH_2_ deformation, indicating strong coupling between the organic cation and the lattice water molecule. The band located at 1582 cm^−1^ is assigned to C=C stretching vibrations within the aromatic ring together with CH_2_ bending contributions. The features at 1491 cm^−1^ and 1453 cm^−1^ correspond to CH_3_ wagging and twisting modes, characteristic of the ethyl substituent. The skeletal vibration of the organic framework appears at 1168 cm^−1^, attributed to C–C stretching, while the band at 1017 cm^−1^ corresponds to NH_2_ rocking vibrations. Additional deformation modes are observed at 941 cm^−1^ and 849 cm^−1^, assigned to C–H bending and out-of-plane aromatic C–H deformation, respectively. The bands located at 754 cm^−1^ and 668 cm^−1^ are associated with water wagging and bending modes, confirming the presence of crystallization water in the lattice. Finally, the feature at 556 cm^−1^ corresponds to a C–C–C skeletal deformation, reflecting collective motions within the organic framework.

#### 3.2.2. [SbI_4_]^−^ vibrational modes

The Raman spectrum provides important information about the vibrational modes of the inorganic [SbI_4_]^−^ anionic unit and the lattice dynamics. The symmetric Sb–I stretching vibration appears as a Raman band at 159 cm^−1^, while the asymmetric Sb–I stretching mode predicted by DFT at approximately 293 cm^−1^ could not be clearly observed experimentally due to the strong photoluminescence background generated by the compound during Raman measurements. Nevertheless, its presence is confirmed by the theoretical calculations reported in Table 1.S. The bands detected at 108 cm^−1^ and 86 cm^−1^ are attributed to asymmetric and symmetric bending modes of the I–Sb–I linkage, characteristic of tetraiodoantimonate units. In the very low-frequency region, the feature observed near 60 cm^−1^ corresponds to a lattice vibration, involving collective motions of the inorganic anion and the surrounding organic cations within the crystal lattice. These Raman modes confirm the structural integrity of the [SbI_4_]^−^ coordination environment and highlight the vibrational coupling between the inorganic chains and the organic components in the hybrid structure.

### 3.3. Optical study: UV-vis spectroscopy and photoluminescence properties

**Fig. 7:**
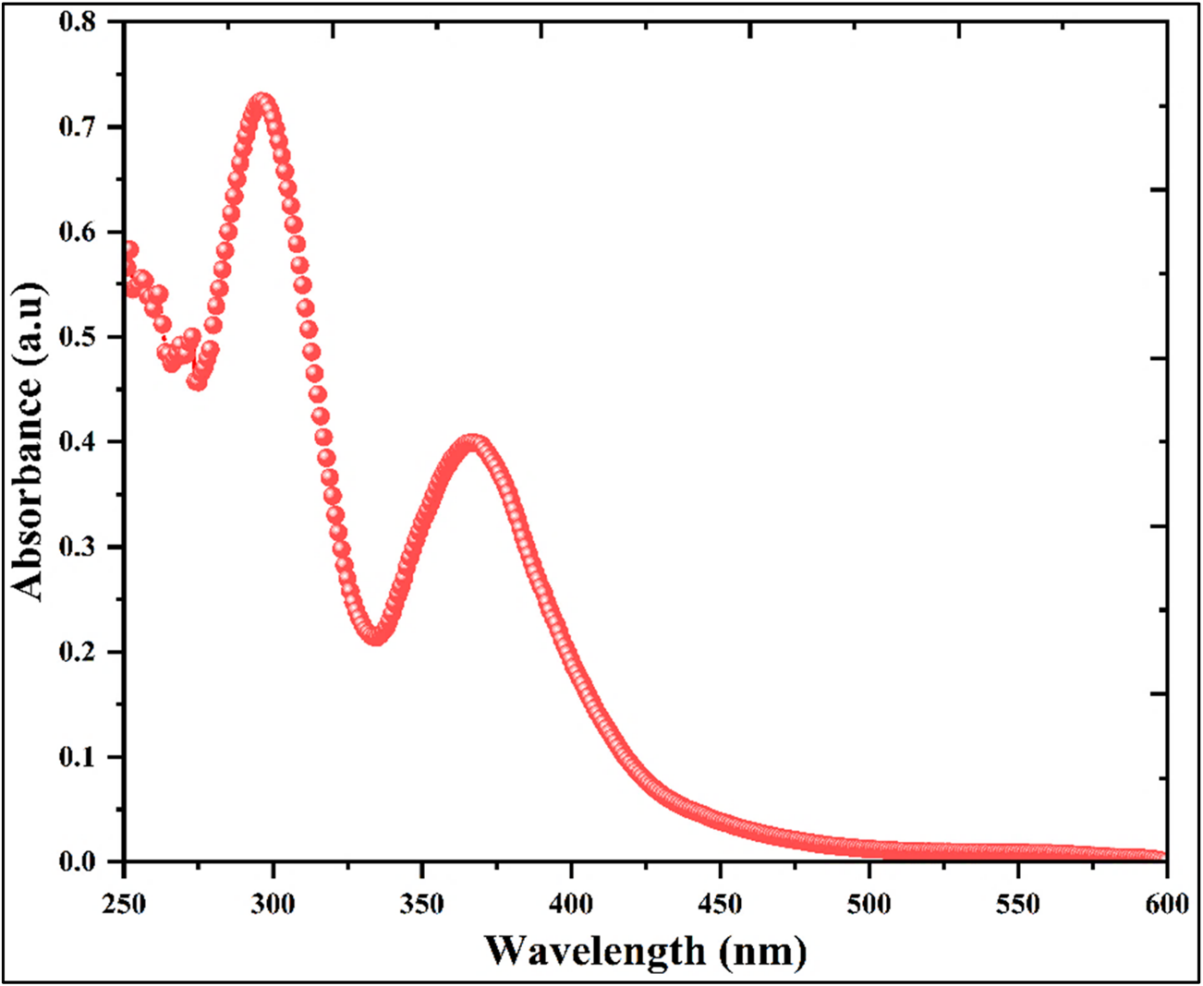
UV-visible optical absorbance.

The optical absorption properties of the hybrid compound (C_10_H_13_N_4_)[SbI_4_]·H_2_O were investigated by UV-visible spectroscopy, and the corresponding absorbance spectrum recorded in aqueous solution is presented in Fig. 7. The spectrum exhibits a continuous absorption profile extending from the ultraviolet region toward the visible range, reflecting electronic transitions occurring within the hybrid system. In hybrid halometallate materials of this type, optical absorption is generally dominated by the inorganic framework, where electronic transitions mainly involve halide p orbitals and metal-centered states associated with the antimony environment. However, it is important to emphasize that the band gap estimated from UV-Vis absorption measurements performed in solution should be considered only as an approximate value. Indeed, the optical response of dissolved species is strongly influenced by several experimental parameters, including temperature, solute concentration, and particularly the nature of the solvent, which can significantly modify the electronic environment of the absorbing species through solvation effects and intermolecular interactions. In the present case, the measurements were carried out in DMSO, a highly polar coordinating solvent that can strongly interact with the hybrid system and alter the observed absorption edge. Consequently, the optical band gap derived from the solution spectrum does not necessarily represent the intrinsic band gap of the crystalline material. A qualitative indication of the actual band gap range can already be inferred from the macroscopic appearance of the crystals shown previously in Fig. 1, which exhibit a relatively dark red coloration, suggesting an optical gap closer to the visible region, likely around ∼2.2 eV. This observation indicates that the optical absorption measured in solution is significantly affected by solvent–solute interactions. For this reason, a more reliable determination of the intrinsic band gap will be performed in a subsequent stage of this work using diffuse reflectance spectroscopy (DRS) on the solid-state material, together with additional complementary investigations aimed at establishing the accurate optical parameters and electronic structure of this compound. Therefore, in the present study, the UV-Visible measurement mainly serves to illustrate the influence of the solvent environment on the optical response of the hybrid material rather than to provide the definitive band gap value.

**Fig. 8:**
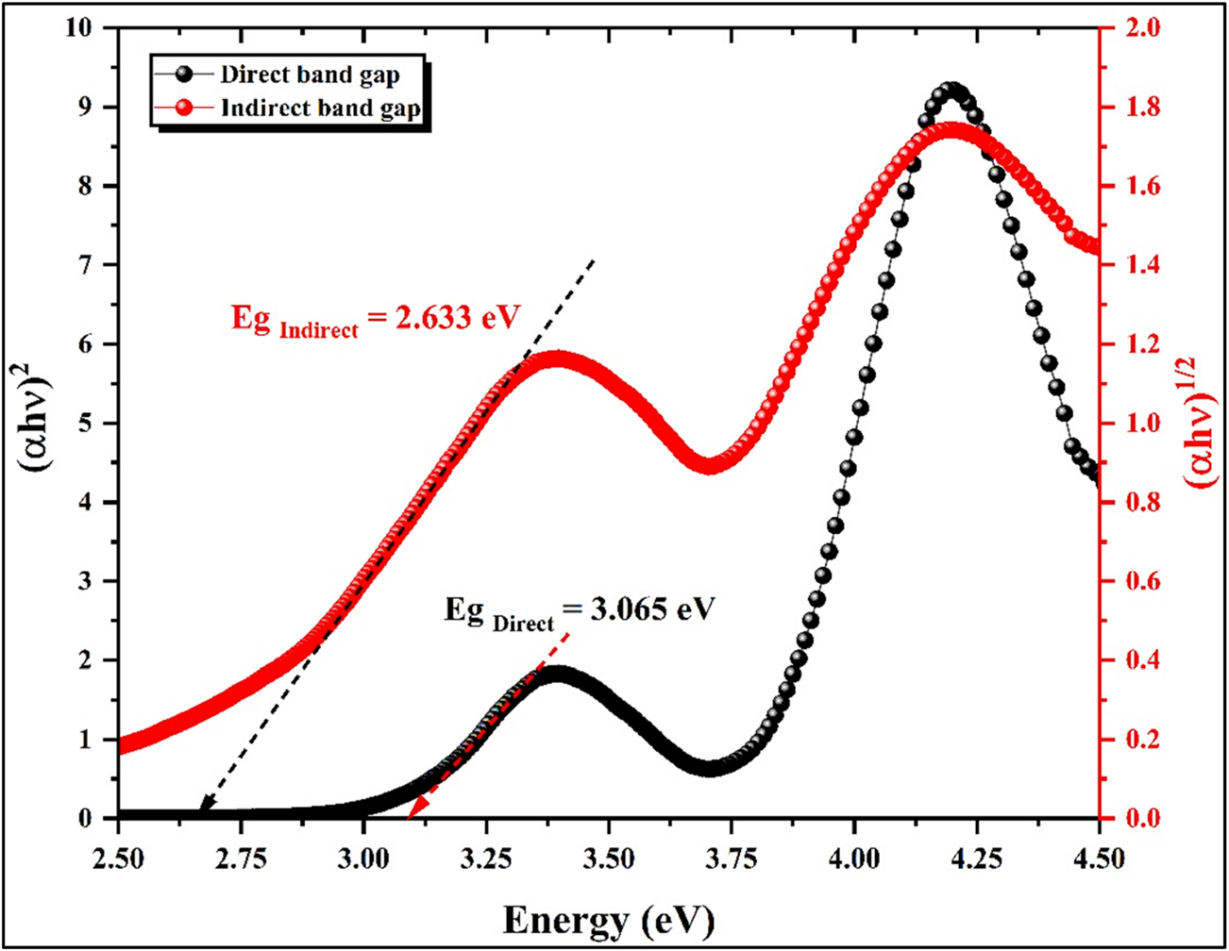
Direct *vs.* indirect transitions Tauc plots.

To further evaluate the optical transition behavior of the hybrid compound (C_10_H_13_N_4_)[SbI_4_]·H_2_O, the absorption data obtained from the UV-Visible spectrum were analyzed using the Tauc equation,^38^ and the corresponding plots for direct and indirect transitions are presented in Fig. 8. The optical band gap was estimated by extrapolating the linear regions of the plots of (*αhν*)^2^ and (*αhν*)^1/2^*versus* the photon energy *hν*, corresponding to the direct and indirect transition models, respectively. From these representations, the extrapolated values suggest an apparent optical gap of approximately 3.065 eV for the direct transition and a lower value for the indirect transition. Nevertheless, as previously discussed, these values must be interpreted cautiously because the measurements were performed in solution, where solvent–solute interactions can substantially perturb the electronic structure and shift the absorption edge.

Despite this limitation, several observations suggest that the direct transition model is the more plausible description of the electronic transition in this compound. Antimony-based halometallate frameworks frequently exhibit direct band-gap characteristics due to the electronic configuration and orbital hybridization of Sb and halide atoms. Moreover, the photoluminescence behavior of the material, discussed below, shows an emission maximum at an energy lower than the absorption-derived band gap together with a relatively sharp emission profile, features typically associated with fluorescence processes occurring in direct band-gap systems. These considerations indicate that the transition represented by the direct Tauc plot is the most reasonable approximation under the present experimental conditions, giving an estimated value of ∼3.065 eV. However, it should be emphasized that this value is expected to be influenced by the solvent environment and therefore does not necessarily correspond to the intrinsic band gap of the crystalline material. A more reliable comparison can be made with the theoretical electronic structure analysis presented in Fig. 10, where the density-of-states calculation predicts an electronic gap of approximately 2.9 eV, suggesting that the true solid-state band gap is likely smaller than the value derived from the solution UV-Vis measurement.

**Fig. 9:**
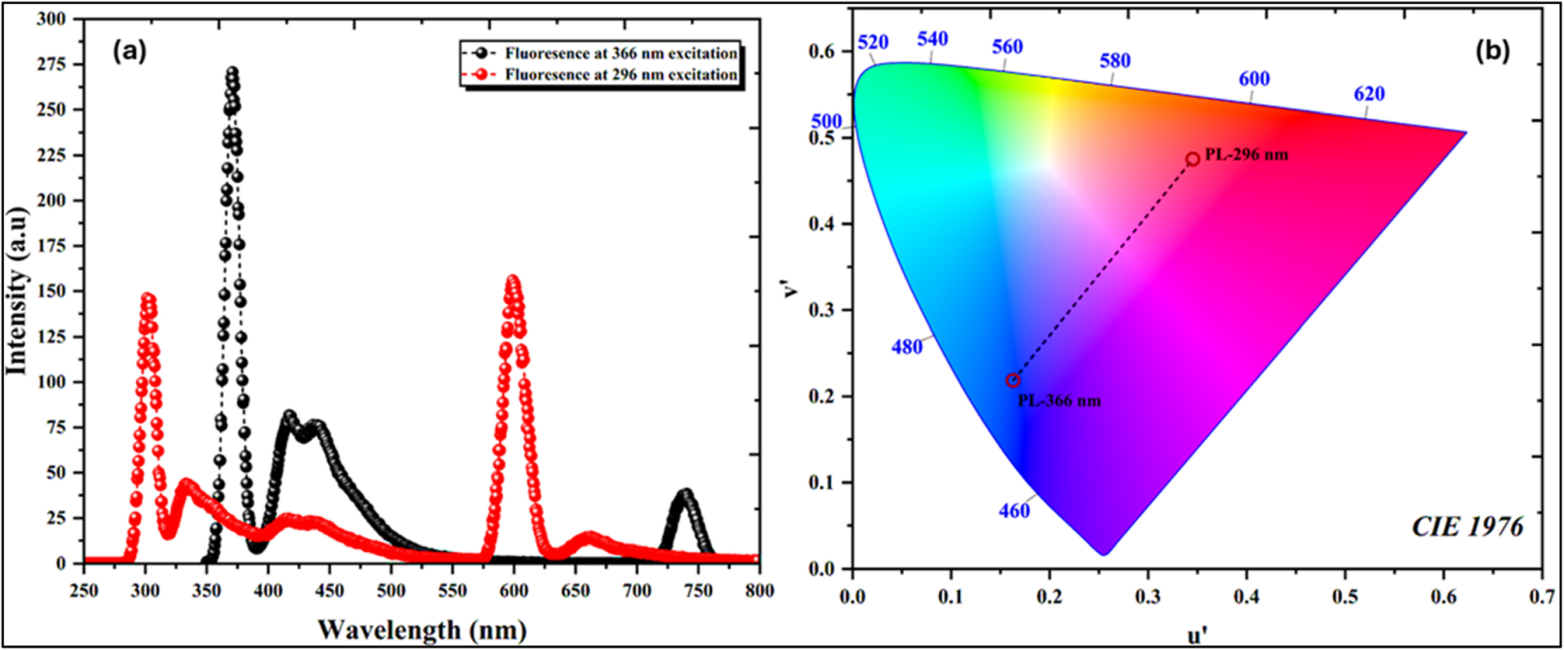
(a) Emission plot of photoluminescence and (b) CIE 1976 chromaticity diagram.

The emission behavior of the hybrid compound (C_10_H_13_N_4_)[SbI_4_]·H_2_O was further examined by photoluminescence spectroscopy, and the corresponding spectra together with the chromaticity coordinates are presented in Fig. 9. The emission spectra were recorded under two different excitation wavelengths, 296 nm and 366 nm, allowing evaluation of the luminescence response of the material under distinct excitation energies. In both cases, the compound exhibits a clear emission band located at a lower energy than the optical absorption edge, confirming that the observed emission originates from a fluorescence process associated with electronic transitions within the hybrid framework. The emission profile is characterized by a relatively sharp dominant peak, particularly evident under 366 nm excitation, indicating a well-defined radiative recombination pathway. Such a sharp emission band is typically characteristic of direct band-gap semiconducting materials, where electron–hole recombination occurs efficiently without requiring phonon-assisted processes. This observation therefore supports the interpretation proposed from the Tauc analysis that the compound most likely exhibits a direct electronic transition.

The presence of two excitation wavelengths also reveals a certain dependence of the emission intensity and spectral distribution on the excitation energy, which can be attributed to the involvement of different excited states or electronic transitions associated primarily with the inorganic [SbI_4_]^−^ framework. In hybrid halometallate systems, photoluminescence is generally governed by the metal–halide network, where electronic transitions typically involve halide p orbitals forming the valence band and metal-centered orbitals contributing to the conduction band. The emission observed here, therefore, likely originates from recombination processes localized within the antimony–iodide framework, while the organic cation mainly contributes to structural stabilization rather than directly participating in the frontier electronic states.

**Fig. 10:**
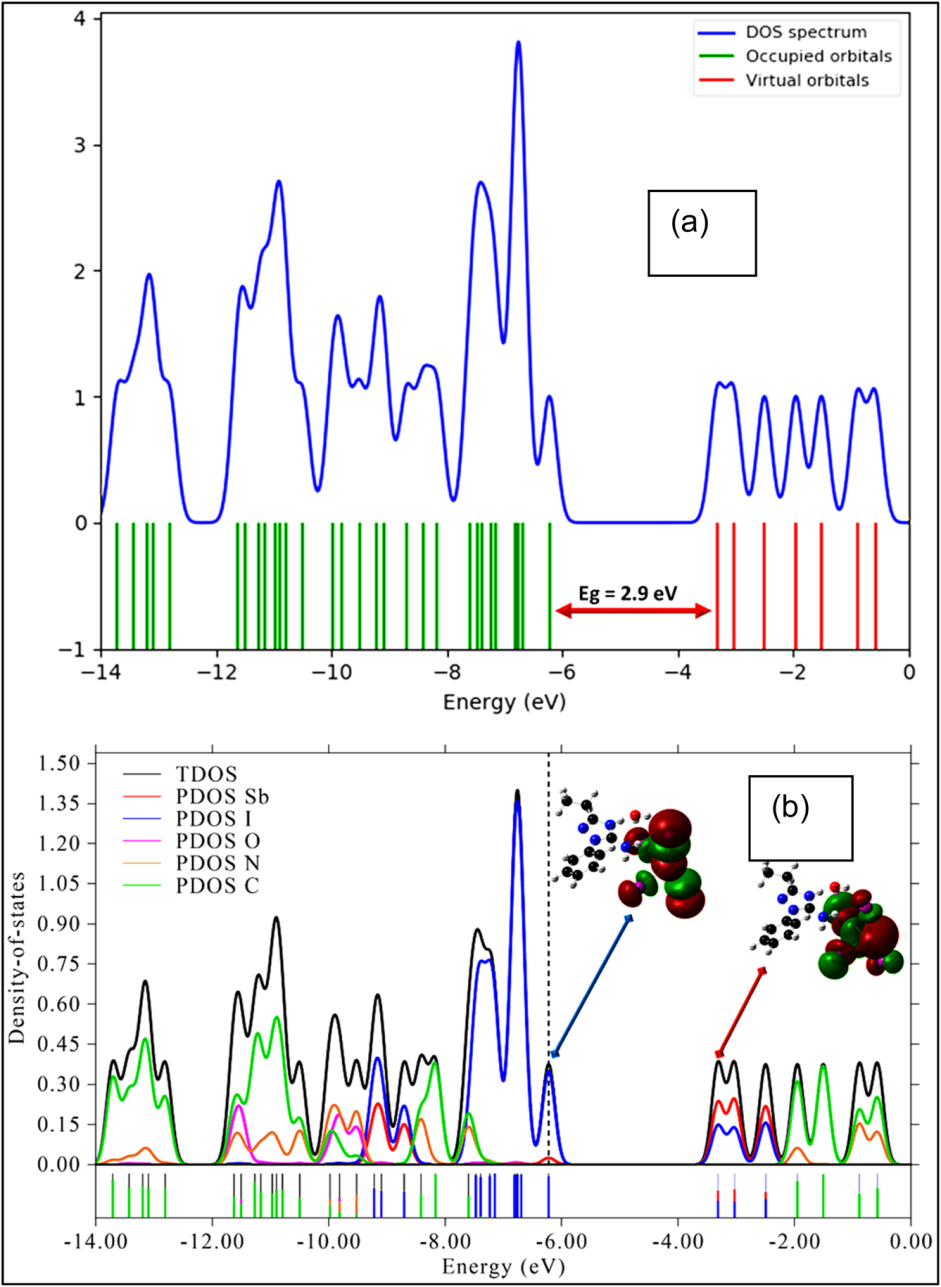
(a) Total density of states (DOS) and (b) projected density of states (PDOS) of the compound.

The chromatic properties of the emitted light were evaluated using the CIE 1976 chromaticity diagram, shown in Fig. 9(b). The calculated chromaticity coordinates indicate that the emitted light falls within the colored region of the diagram, confirming the visible photoluminescence of the compound. This luminescent behavior, combined with the semiconducting band structure suggested by both optical measurements and the theoretical DOS analysis presented in Fig. 10, highlights the potential of this hybrid antimony–iodide material for optoelectronic applications where controlled emission and photon–matter interactions are required.

### 3.4. Theoretical investigation

#### 3.4.1. Frontier molecular orbital (FMO) analysis

The Density of States (DOS) and Partial Density of States (PDOS) analyses provide key insights into the electronic structure of the (C_10_H_13_N_4_)[SbI_4_]·H_2_O hybrid material, revealing the characteristics of frontier orbitals, band dispersion, and elemental contributions to electronic states. The DOS spectrum (Fig. 10(a)) illustrates the distribution of electronic states across energy levels, while the PDOS spectrum (Fig. 10(b)) identifies the orbital contributions, particularly those associated with the HOMO and LUMO levels, enabling a detailed interpretation of orbital hybridization and charge distribution.

The total DOS indicates a well-defined bandgap with clearly separated valence band maximum (VBM) and conduction band minimum (CBM). The energy difference between these states corresponds to the HOMO–LUMO gap, indicating semiconducting behavior. This moderate bandgap governs the material's optoelectronic properties, including optical absorption, emission, and charge transport. The absence of mid-gap states suggests electronic purity and a reduced probability of defect-related charge trapping or recombination.

The PDOS spectrum (Fig. 10(b)) reveals the orbital composition of the electronic bands. The valence band is mainly dominated by iodine (I) 5p orbitals, which strongly contribute to the states near the VBM due to iodine's high polarizability and its key role in bonding within the 1D [SbI_4_]^*n*−^ chain. Contributions from Sb 5 s and 5p orbitals are also observed, indicating moderate Sb–I orbital hybridization that stabilizes the anionic framework. In contrast, the conduction band is primarily governed by Sb 5p orbitals, showing that the lowest unoccupied states are mainly localized on the antimony center. This metal-centered conduction band is typical of halide-based hybrid materials.

The organic cation (C_10_H_13_N_4_)^+^ contributes only weakly to the frontier orbitals, consistent with many hybrid systems where the inorganic sublattice controls the principal electronic transitions. Minor contributions from nitrogen and carbon p orbitals suggest limited interactions with the inorganic framework through weak hydrogen bonding or electrostatic effects that may slightly influence band alignment. Consequently, the electronic states associated with the 1D [SbI_4_]^*n*−^ chain dominate optical absorption and charge carrier dynamics.

The DOS curvature near the band edges also provides insight into carrier transport properties. Steep DOS features around the VBM and CBM indicate relatively low effective masses for charge carriers, which may enhance charge mobility. Additionally, the asymmetric DOS distribution near the band edges suggests an indirect bandgap character, consistent with the UV-Vis absorption results that indicate an indirect transition. Such a band structure may influence radiative recombination processes and could favor applications such as photodetection or nonlinear optical systems.

Overall, the DOS and PDOS analyses demonstrate that the electronic behavior of (C_10_H_13_N_4_)[SbI_4_]·H_2_O is primarily governed by the inorganic [SbI_4_]^*n*−^ chain, where iodine p orbitals dominate the valence band and antimony p orbitals dominate the conduction band. The organic cation plays a secondary role, mainly affecting structural stability and weak electronic interactions. The well-defined bandgap and absence of defect states indicate promising electronic characteristics for potential optoelectronic applications.

#### 3.4.2. MEP, NCI-RDG, ELF, and LOL analyses

A comprehensive theoretical investigation of the (C_10_H_13_N_4_)[SbI_4_]·H_2_O hybrid material involves multiple electronic structure analyses, each providing unique insights into different aspects of intermolecular interactions, charge localization, and bonding characteristics. The Molecular Electrostatic Potential (MEP) analysis reveals the charge distribution across the molecular framework, identifying regions of electrophilic and nucleophilic character that influence hydrogen bonding and supramolecular organization. The Non-Covalent Interaction (NCI-RDG) analysis visualizes the weak intermolecular forces that govern lattice stability, highlighting the role of van der Waals interactions, halogen bonding, and π-stacking. The Electron Localization Function (ELF) and Localized Orbital Locator (LOL) analyses offer a deeper understanding of electronic delocalization and orbital interactions, shedding light on the extent of electron pairing and covalency within the system. Together, these computational techniques provide a holistic picture of the electronic environment, enabling a deeper interpretation of the material's stability, electronic structure, and potential functional applications.

##### 3.4.2.1. Molecular electrostatic potential MEP analysis

The Molecular Electrostatic Potential (MEP) analysis provides key insight into the charge distribution and electrostatic interactions within the (C_10_H_13_N_4_)[SbI_4_]·H_2_O hybrid material, identifying electron-rich and electron-deficient regions that govern reactivity and intermolecular interactions. The MEP surface (Fig. 4.S) is color-coded: red regions indicate electron-rich, nucleophilic zones, while blue regions correspond to electron-deficient, electrophilic areas.

The most negative regions are localized on the halide atoms (iodine), reflecting their high electronegativity and polarizability. These sites serve as primary centers for electrostatic attractions, including halogen and hydrogen bonding. The most positive regions are centered on the antimony (Sb) atom, highlighting its electropositive character and its role as a coordination hub within the [SbI_4_]^−^ anionic unit.

The organic cation (C_10_H_13_N_4_)^+^ exhibits a distributed electrostatic potential, with moderately positive areas around the protonated nitrogen atoms, confirming their involvement in hydrogen bonding with both the anionic framework and the crystallized water molecule. The water molecule further modulates the local potential, enhancing hydrogen-bond stabilization within the lattice. Overall, the MEP topology aligns with the observed supramolecular assembly, demonstrating that electrostatic complementarity underpins the stable 3D network.

Additionally, the MEP surface suggests charge transfer dynamics: the potential gradient between halide-rich anions and the cationic framework indicates internal electrostatic stabilization that could influence charge separation and transport. The pronounced contrast between electron-rich and electron-deficient regions implies a strong internal dipole moment, potentially affecting the material's dielectric properties, polarization behavior, and overall electronic and optical performance.

##### 3.4.2.2. Non-covalent interaction NCI-RDG analysis

The Non-Covalent Interaction (NCI) analysis, using the Reduced Density Gradient (RDG) approach, provides a detailed visualization of weak interactions governing the stability and organization of the (C_10_H_13_N_4_)[SbI_4_]·H_2_O hybrid material. This technique highlights van der Waals forces, hydrogen bonding, halogen bonding, and steric repulsions, which collectively shape the supramolecular assembly. Fig. 5.S shows the NCI-RDG isosurface mapped onto the molecular framework, where deep blue indicates strong attractive interactions (hydrogen bonds), green represents moderate van der Waals forces, and red highlights repulsive steric interactions.

The analysis shows that hydrogen bonding is the primary stabilizing force, evidenced by intense blue regions between the protonated nitrogen atoms of the organic cation and the crystallized water molecule, which bridges molecular components to reinforce 3D lattice cohesion. Halogen–halogen interactions are observed as elongated green isosurfaces between adjacent iodine atoms, reflecting Type-II halogen bonding. These interactions maintain the spatial separation of the anionic [SbI_4_]^−^ units while preventing covalent overlinking, ensuring the structural integrity of the 1D framework.

Van der Waals interactions pervade the organic sublattice, particularly within π-stacking regions of the aromatic cations. These dispersive forces enhance packing efficiency, minimize voids, and stabilize the lattice through π-electron cloud overlap without introducing steric strain. Red regions denote steric repulsions, mainly around the Sb coordination sphere, where the bulky [SbI_4_]^−^ unit prevents excessive compression and maintains molecular separation.

Overall, the NCI-RDG analysis reveals the delicate balance of forces stabilizing the hybrid material. Hydrogen bonding dominates, supplemented by halogen bonding that preserves anionic separation, while van der Waals forces organize the organic cations. Steric repulsions act as a regulatory mechanism, preventing overpacking and maintaining equilibrium among non-covalent interactions. This comprehensive view of intermolecular forces provides critical insights into the structural stability, electronic environment, and potential functional behavior of hybrid materials, highlighting how directional and non-directional interactions collectively dictate lattice cohesion and supramolecular architecture.

##### 3.4.2.3. Electron localization function (ELF) and localized orbital locator (LOL) analyses

Fig. 6.S and 7.S present a detailed analysis of the Electron Localization Function (ELF) and Localized Orbital Locator (LOL) in the (C_10_H_13_N_4_)[SbI_4_]·H_2_O hybrid material, visualizing electronic localization, bonding interactions, and charge delocalization. Fig. 6.S establishes the three principal planes (*XY*, *XZ*, *YZ*), chosen to dissect the electronic landscape and enable clear attribution of ELF and LOL features. Fig. 7.S maps the ELF and LOL distributions on these planes, revealing the electronic organization of the material.

The ELF analysis shows electron-pairing tendencies, with values ranging from 0 (delocalized) to 1 (highly localized). The Sb(iii) center in the 1D [SbI_4_]^*n*−^ chain exhibits an asymmetric ELF, confirming a stereochemically active lone pair that induces distortions from ideal tetrahedral geometry, producing the observed seesaw-like coordination. ELF maps along the *XY* and *XZ* planes highlight the polarization of Sb–I bonds, with electron density skewed toward iodine, indicating predominantly ionic character. The *YZ* plane visualizes halogen–halogen interactions, where weakly shared density contributes to non-covalent stabilization of the extended anionic chain.

Within the organic cation, ELF shows high localization around nitrogen lone pairs engaged in hydrogen bonding with the anionic framework, reinforcing lattice cohesion. The aromatic rings exhibit delocalized electron density, consistent with π-conjugation that supports π–π stacking and structural stability.

LOL analysis complements ELF by revealing orbital-based charge transport and delocalization. Localized orbitals dominate Sb–I bonds, confirming mixed ionic-covalent character, while iodine regions show low LOL, reflecting weak intermolecular interactions. The cationic aromatic system exhibits smoothly varying LOL, highlighting π-electron delocalization that aids overall charge distribution, even without direct involvement in frontier orbital transitions.

Integrating ELF and LOL across the three planes demonstrates clear electronic separation: the 1D anionic framework governs charge localization and band structure, while the cation stabilizes *via* electrostatic and dispersive forces. ELF identifies localized charge centers and lone pairs, whereas LOL highlights orbital delocalization trends relevant to electronic and optoelectronic behavior.

In summary, Fig. 6.S and 7.S illustrate the intricate electronic interplay in (C_10_H_13_N_4_)[SbI_4_]·H_2_O. The Sb(iii) lone pair drives anionic distortions, Sb–I bonding and secondary halogen interactions stabilize the 1D framework, and hydrogen bonding, along with π-electron delocalization in the cation, maintain structural integrity and charge balance, producing a harmonized electronic environment essential for the material's functional properties.

### 3.5. Hirshfeld surface analysis (HSA)

**Fig. 11:**
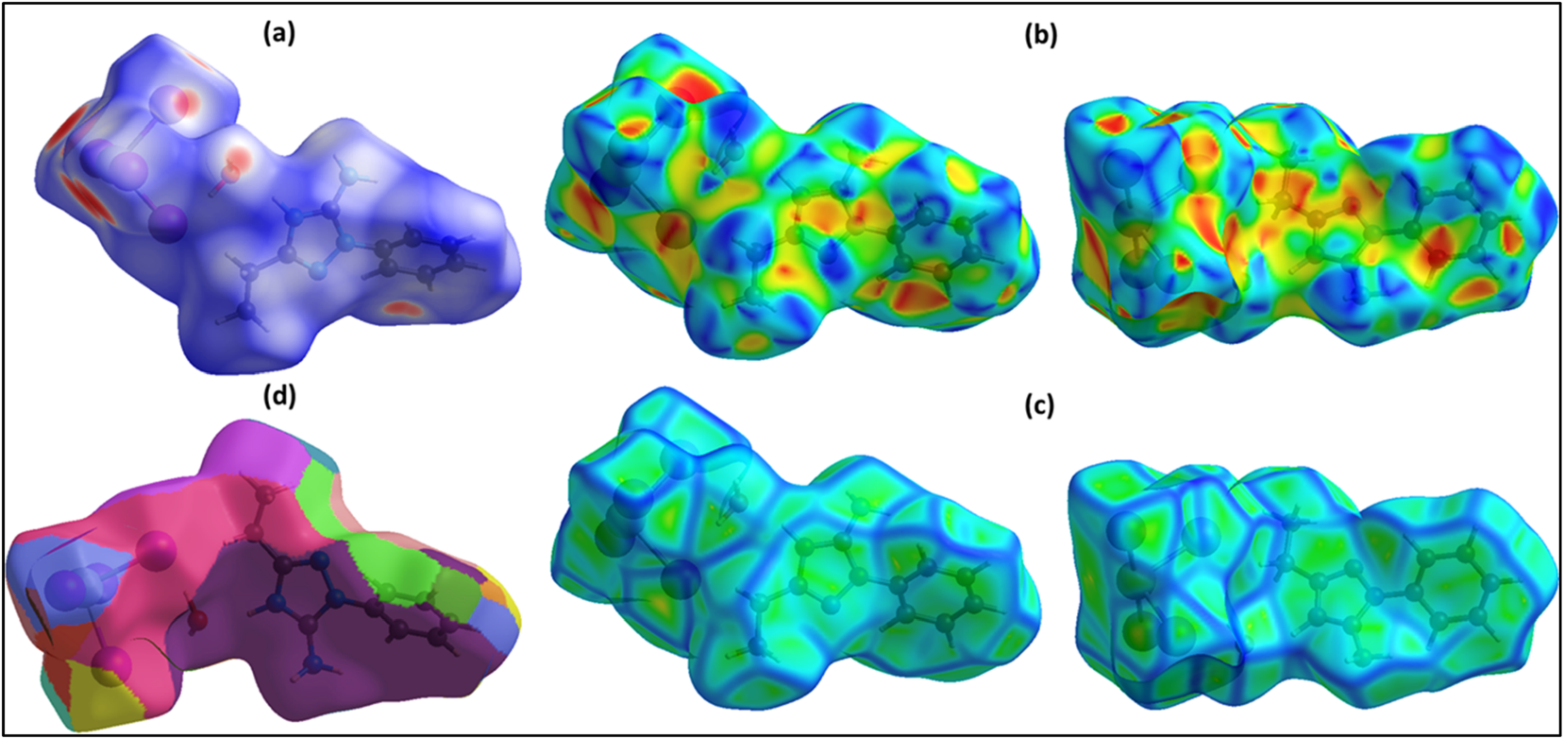
(a) Hirshfeld surface, (b) shape index, (c) curvedness, (d) fragment patches.

Fig. 11 presents a comprehensive Hirshfeld surface (HS) analysis of the (C_10_H_13_N_4_)[SbI_4_]·H_2_O hybrid material, visualizing intermolecular interactions, molecular shape, and the role of π-stacking in the structural framework. The analysis is divided into four subfigures: (a) the total HS mapped with dnorm, (b) the shape index highlighting π–π stacking, (c) the curvedness parameter supporting shape index attributions, and (d) the fragment patch, showing contributions from neighboring molecules.

The dnorm surface (Fig. 11(a)) provides a global view of close-contact interactions. Red regions indicate strong interactions, white denotes contacts near van der Waals distances, and blue reflects weak or absent interactions. Prominent red spots appear at hydrogen bonding sites between the cation, anionic chain, and crystallized water, confirming hydrogen bonding as the main stabilizing force, consistent with MEP and NCI-RDG results. Minimal red regions between anionic units show that the 1D [SbI_4_]^*n*−^ chain remains extended, not forming isolated units.

The shape index (Fig. 11(b)) identifies complementary concave (red) and convex (blue) regions corresponding to overlapping π-systems, confirming that both aromatic rings participate in π–π stacking. These interactions form an alternating network that extends through the lattice, optimizing dispersive forces, minimizing steric hindrance, and reinforcing organic sublattice cohesion.

Curvedness analysis (Fig. 11(c)) validates π–π stacking by highlighting planarity: low curvature indicates flat surfaces favoring π-stacking, while high curvature reflects steric limitations. Both views align with the shape index, confirming the engagement of both aromatic rings in well-defined π-stacking interactions, contributing to lattice stability.

The fragment patch (Fig. 11(d)) decomposes surface contributions from neighboring molecules, enabling quantitative assessment of interaction zones. It distinguishes hydrogen bonding, halogen bonding, van der Waals interactions, and π–π stacking. The cation's interaction profile shows a balanced combination of hydrogen bonding and π–π dispersive interactions, with the latter crucial for supramolecular assembly. The fragment patch also confirms that the 1D [SbI_4_]^*n*−^ chain connectivity is continuous rather than fragmented.

**Fig. 12:**
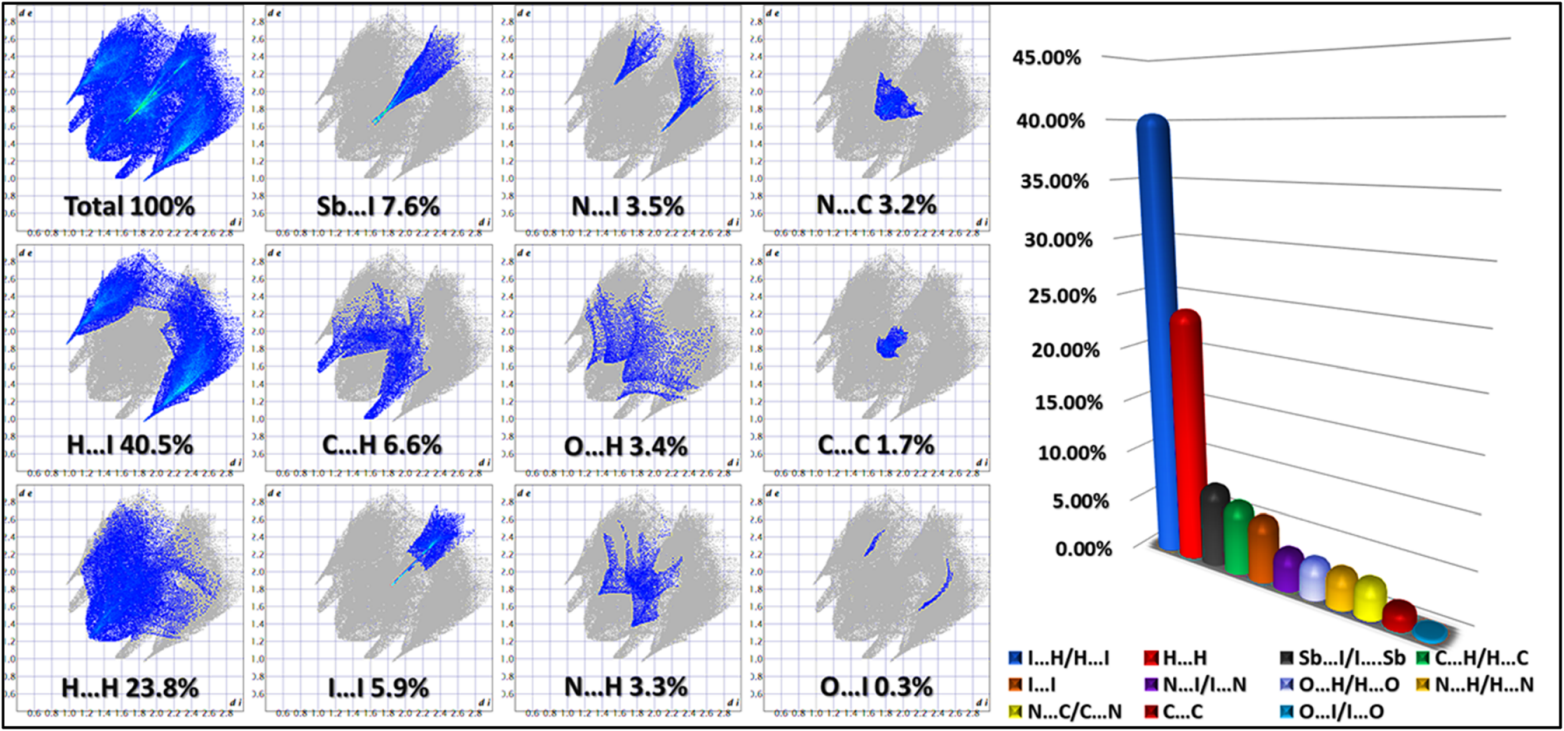
2D fingerprints of the intermolecular interactions.

Fig. 12 presents a detailed quantitative analysis of intermolecular interactions in the (C_10_H_13_N_4_)[SbI_4_]·H_2_O hybrid material through Hirshfeld surface decomposition and 2D fingerprint plots, providing statistical insight into the contributions of various non-covalent forces to crystal stability. The Hirshfeld decomposition isolates specific interaction types, while the 2D fingerprint plots visually map interatomic distances, distinguishing hydrogen bonding, halogen bonding, van der Waals forces, and π–π stacking contributions.

The decomposition confirms hydrogen bonding as the principal stabilizing interaction. N–H⋯I, O–H⋯I, and N–H⋯O contacts dominate the surface coverage, with H⋯I interactions being the most prominent. These strong electrostatic contacts connect the organic cation to the [SbI_4_]^*n*−^ chain, while O–H⋯I interactions involving the crystallized water molecule bridge lattice components, reinforcing overall structural integrity. The 2D fingerprint plots reveal sharp spikes at short donor–acceptor distances, indicating highly directional hydrogen bonds with well-defined geometries that enhance lattice robustness.

Moderate van der Waals contributions are evident in the mid-region of the fingerprint plots, corresponding to dispersive H⋯H interactions and supporting π–π stacking within the cationic framework. These interactions, though weaker than hydrogen bonding, are crucial for efficient molecular packing and lattice cohesion. The broad, scattered mid-region of the plots reflects their distributed and less directional nature, complementing stronger electrostatic contacts.

Halogen bonding interactions, particularly I⋯I and I⋯H, are observed as elongated features in the fingerprint plots. These secondary interactions stabilize the 1D [SbI_4_]^*n*−^ chain without forming covalent bonds, fine-tuning anionic sublattice organization. Although their contribution is smaller than hydrogen bonding, their consistent presence indicates a non-negligible role in maintaining structural hierarchy and spatial separation of the inorganic framework.

In summary, the Hirshfeld surface decomposition and 2D fingerprint analyses quantitatively confirm the hierarchy of intermolecular interactions in (C_10_H_13_N_4_)[SbI_4_]·H_2_O: hydrogen bonding dominates lattice stabilization, van der Waals and π–π stacking interactions organize the cationic framework, and halogen bonding subtly reinforces the anionic sublattice. These findings provide a precise and comprehensive understanding of the non-covalent forces shaping the supramolecular architecture and overall crystal stability.

The final breakdown of interaction percentages from the Hirshfeld surface decomposition provides a hierarchical view of stabilizing forces, revealing the following trends:

H⋯I hydrogen bonding interactions dominate, acting as the primary electrostatic stabilizers.

H⋯O interactions contribute to additional lattice cohesion, particularly *via* the crystallized water molecule.

I⋯I halogen interactions provide secondary stabilization, ensuring the spatial connectivity of the 1D anionic chain.

H⋯H van der Waals interactions fine-tune molecular packing, reducing void space and enhancing dispersion-driven lattice reinforcement.

Fig. 8.S and 9.S collectively provide a comprehensive quantitative and energetic characterization of the (C_10_H_13_N_4_)[SbI_4_]·H_2_O hybrid material, reinforcing the understanding of its supramolecular organization and crystal stability.

The void space analysis in Fig. 8.S reveals a calculated unoccupied fraction of 9.788%, indicating a densely packed lattice where intermolecular forces efficiently minimize empty regions. This low void fraction reflects an optimized balance between structural rigidity and molecular flexibility, ensuring that hydrogen bonding and π–π stacking interactions are maximized to stabilize the lattice without introducing excessive strain. The absence of significant channels or solvent-accessible cavities confirms that the 1D [SbI_4_]^*n*−^ chains remain continuous and structurally coherent, while the organic cationic network is tightly interlocked. From a functional perspective, such compact packing limits defect formation, reduces charge localization in void regions, and enhances dielectric uniformity, all of which are beneficial for potential electronic and optoelectronic applications.

Fig. 9.S provides an energy framework analysis calculated with CrystalExplorer 21.5 at the HF/3-21G level, decomposing intermolecular interactions into electrostatic (*E*_ele_), polarization (*E*_pol_), dispersion (*E*_dis_), repulsion (*E*_rep_), and total interaction energy (*E*_tot_). The numerical evaluation shows that electrostatic interactions dominate the lattice stabilization, with pairwise *E*_ele_ values around −150 kJ mol^−1^, corresponding to strong coulombic attractions between the cationic (C_10_H_13_N_4_)^+^ units, the anionic [SbI_4_]^−^ chains, and the crystallized water molecules. These strong, attractive interactions are moderated by repulsive terms (*E*_rep_ ≈ +301 kJ mol^−1^), producing moderate total interaction energies (*E*_tot_ ≈ −50 to −51 kJ mol^−1^), indicative of a highly optimized balance between attraction and steric repulsion that maintains lattice integrity without inducing excessive strain.

The dispersion contributions (*E*_dis_ ≈ −36 to −37 kJ mol^−1^) are associated with π–π stacking between aromatic rings of the organic cations, as well as I⋯I and I⋯H contacts within the inorganic framework. Although secondary in magnitude, these dispersive interactions are crucial for reinforcing lattice cohesion, complementing the dominant electrostatic and hydrogen bonding forces. Polarization effects (*E*_pol_) remain minor, consistent with the predominantly ionic and hydrogen-bond-driven character of the lattice. The isotropic distribution of interaction energies further indicates that the lattice stabilization is cooperative and hierarchical, without privileging a single crystallographic direction, despite the 1D nature of the anionic chains.

Together, the void-space and energy analyses quantitatively and energetically validate the supramolecular organization of (C_10_H_13_N_4_)[SbI_4_]·H_2_O. The low void fraction confirms dense packing driven by directional hydrogen bonds and π–π interactions. At the same time, the energy decomposition highlights the predominance of electrostatics, supplemented by dispersion and minor polarization, in maintaining lattice robustness. This integrated description corroborates the hierarchical, highly optimized network of non-covalent interactions identified in previous Hirshfeld surface, fingerprint, and electronic analyses, demonstrating a structurally and energetically stable hybrid crystal lattice suitable for functional applications.

### 3.6. Thermal analyses: TGA and DSC

**Fig. 13:**
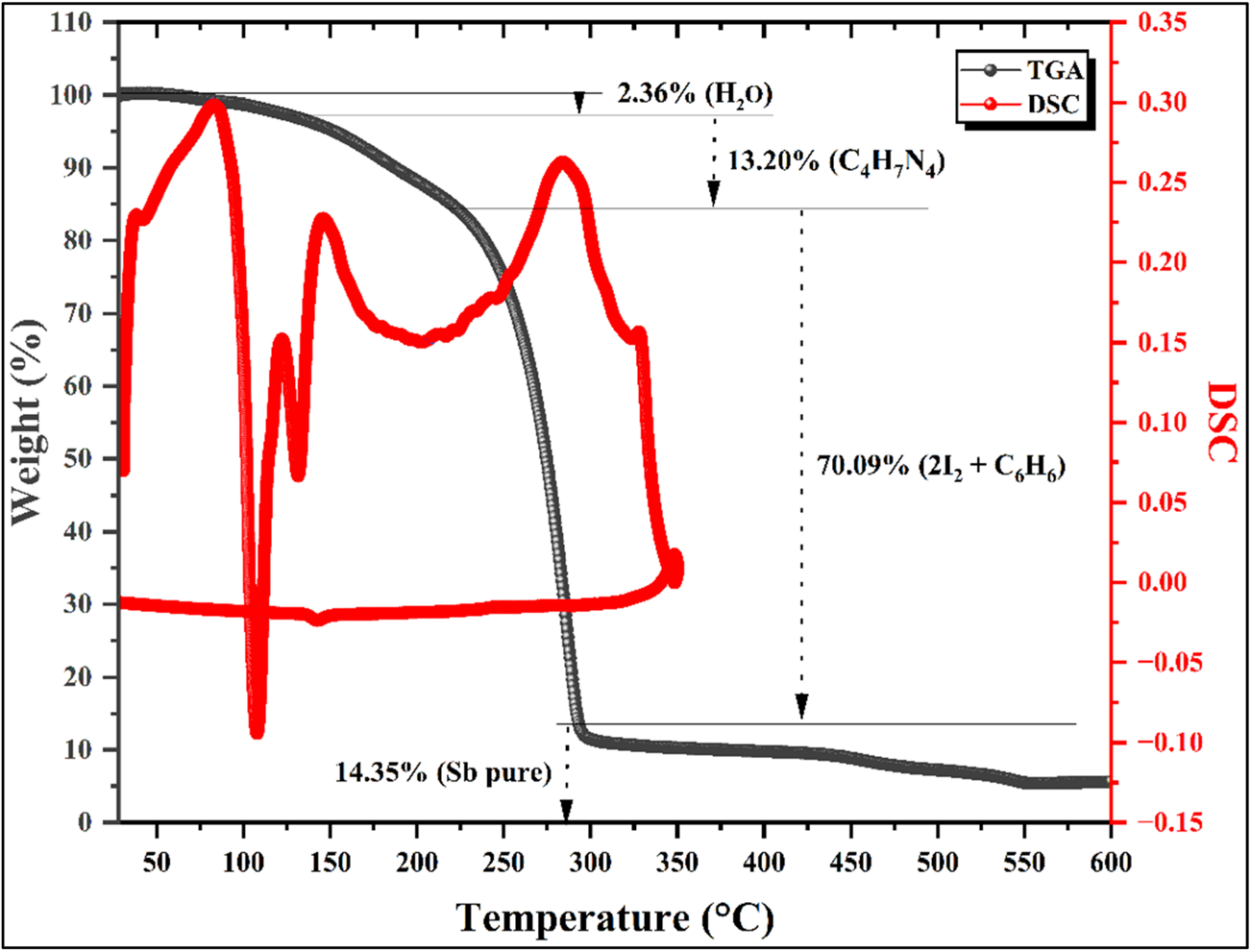
Thermogravimetric analysis (TGA) of the titled compound.

The thermal stability and decomposition behavior of (C_10_H_13_N_4_)[SbI_4_]·H_2_O were examined by simultaneous thermogravimetric analysis (TGA) and differential scanning calorimetry (DSC) (Fig. 13), allowing a detailed correlation between mass-loss events and their thermal signatures. With a molar mass of 836.6 g mol^−1^, the compound exhibits a well-resolved, multistep decomposition profile, in which each thermal event can be rationally assigned based on interaction strength, bond hierarchy, and excellent agreement between theoretical and experimental mass losses.

The first thermal event, centered at approximately 110 °C, corresponds unambiguously to the release of the crystallized water molecule. This species is not covalently bonded to the framework and is retained solely through hydrogen bonding and other weak intermolecular interactions, making it the most labile component of the structure. The process is accompanied by a sharp endothermic peak in the DSC curve, reflecting the energy required to disrupt the stabilizing non-covalent interactions and to promote the water molecule to a higher-energy, mobile state prior to its elimination. The experimental mass loss of 2.36% closely matches the theoretical value of 2.15%, with a deviation of only 0.16%, confirming the presence of one lattice water molecule per formula unit and marking the upper temperature limit for preservation of the complete crystal architecture.

Upon dehydration, the compound undergoes a progressive degradation of the organic cation between 110 and 220 °C, manifested as two closely spaced, nearly superposed endothermic features in the DSC trace. The first, a sharp endothermic peak around 130 °C, is attributed to the cleavage of the most thermally labile bonds within the organic moiety, leading to the elimination of small volatile fragments associated with the aliphatic and amino functionalities. The second contribution, observed as a broader endothermic signal extending toward higher temperatures, reflects the gradual breakdown of the five-membered heteroaromatic C_2_N_3_ ring, a process that requires progressively higher energy due to the presence of strong covalent bonds and partial aromatic stabilization. These overlapping events together correspond to the loss of a C_4_H_7_N_4_ fragment, with a theoretical mass loss of 13.30% and an experimental value of 13.20%, demonstrating excellent internal consistency and supporting the proposed stepwise degradation of the organic cation.

A major mass-loss event occurs in the temperature range 220–285 °C, associated with the destruction of the remaining six-membered aromatic framework of the organic residue (benzene, C_6_H_6_), concomitant with the volatilization of halide species from the inorganic sublattice (2 I_2_). This stage appears as a continuation of the preceding endothermic process in the DSC curve, indicating a thermally demanding decomposition that involves the rupture of robust covalent and ionic interactions. The experimental mass loss of 70.09% is in remarkable agreement with the calculated value of 70.00%, consistent with the complete removal of the organic component and of the iodide content, and confirming the extensive breakdown of the hybrid framework.

At temperatures above 285 °C, the TGA curve corresponds to the formation of elemental antimony as the final residue. The residual mass fraction (14.35% experimentally, 14.55% theoretically) closely matches the expected value for Sb, indicating that all organic and halide constituents have been effectively eliminated. This final stage highlights the complete conversion of the hybrid material into its inorganic metallic core under thermal treatment.

Overall, the TGA-DSC analysis reveals a highly coherent and quantitatively consistent decomposition sequence, governed by the progressive disruption of non-covalent interactions, followed by cleavage of covalent bonds within the organic cation, and finally by the collapse of the inorganic halide framework. The excellent agreement between theoretical and experimental mass losses at each stage provides strong validation of the proposed attribution, while the endothermic nature of all major events reflects the energy-intensive breakdown of an initially well-stabilized hybrid crystal lattice.

## 4. Conclusion

In summary, a new one-dimensional antimony-based organic–inorganic hybrid material, (C_10_H_13_N_4_)[SbI_4_]·H_2_O, has been successfully synthesized *via* hydrothermal methods and comprehensively investigated through structural, spectroscopic, thermal, and computational approaches. Single-crystal X-ray diffraction revealed that the structure consists of infinite [SbI_4_]^−^ chains stabilized by protonated organic cations and lattice water molecules through a dense network of hydrogen bonding and non-covalent interactions, which govern the supramolecular architecture and ensure structural stability. Complementary PXRD measurements confirmed the phase purity of the obtained crystals, while Hirshfeld surface and energy framework analyses demonstrated that electrostatic interactions and hydrogen bonding dominate the crystal packing, with dispersion contributions further reinforcing lattice cohesion.

Vibrational analyses using FT-IR and Raman spectroscopy, supported by DFT calculations, provided a consistent interpretation of the molecular and lattice vibrational modes associated with both the organic and inorganic components. Optical investigations revealed visible photoluminescence behavior under UV excitation, indicating efficient radiative recombination processes within the antimony–iodide framework. Although the optical band gap derived from solution UV-Vis spectroscopy (∼3.06 eV) is influenced by solvent effects, theoretical DOS calculations predict an intrinsic band gap of approximately 2.9 eV, confirming the semiconducting nature of the material and highlighting the dominant contribution of iodine p orbitals to the valence band and antimony orbitals to the conduction band. Thermal analysis further demonstrated that the compound exhibits a coherent multistep decomposition pathway with excellent agreement between theoretical and experimental mass losses, confirming the structural composition and stability hierarchy within the hybrid lattice.

Overall, the integrated experimental and theoretical results establish (C_10_H_13_N_4_)[SbI_4_]·H_2_O as a structurally stable one-dimensional hybrid halometallate with tunable electronic and luminescent properties. These findings contribute to the growing family of lead-free antimony-based hybrid materials and highlight their potential for future applications in optoelectronic and photonic technologies.

## Conflicts of interest

The authors declare that they have no known competing financial interests or personal relationships that could have appeared to influence the work reported in this paper.

## Supplementary Material

RA-016-D6RA02020H-s001

RA-016-D6RA02020H-s002

## Data Availability

The raw/processed data required to reproduce these findings are available and can be sent if requested. Supplementary information (SI) is available. See DOI: https://doi.org/10.1039/d6ra02020h. CCDC 2431918 contains the supplementary crystallographic data for this paper.^39^

## References

[cit1] Grätzel M. (2014). Nat. Mater..

[cit2] Snaith H. J. (2013). J. Phys. Chem. Lett..

[cit3] Mitzi D. B. (1999). Prog. Inorg. Chem..

[cit4] Kovalenko M. V., Protesescu L., Bodnarchuk M. I. (2017). Science.

[cit5] Wang Y., Zhang T., Kan M., Zhao Y. (2018). Angew. Chem., Int. Ed..

[cit6] Shi D., Adinolfi V., Comin R. (2015). et al.. Science.

[cit7] Niu G., Guo X., Wang L. (2015). J. Mater. Chem. A.

[cit8] Leng M., Yang Y., Zeng K. (2018). et al.. J. Am. Chem. Soc..

[cit9] Saidaminov M. I., Abdelhady A. L., Murali B. (2015). et al.. Nat. Commun..

[cit10] Xiao Z., Song Z., Yan Y. (2019). Adv. Mater..

[cit11] Kepenekian M., Even J. (2017). J. Phys. Chem. Lett..

[cit12] Brandt R. E., Stevanović V., Ginley D. S., Buonassisi T. (2015). MRS Commun..

[cit13] Filip M. R., Giustino F. (2014). Phys. Rev. B: Condens. Matter Mater. Phys..

[cit14] Yantara N., Bruno A., Milhem S. (2020). et al.. Adv. Funct. Mater..

[cit15] Even J., Pedesseau L., Jancu J. M., Katan C. (2013). J. Phys. Chem. Lett..

[cit16] Yin W. J., Shi T., Yan Y. (2014). Adv. Mater..

[cit17] Stoumpos C. C., Kanatzidis M. G. (2015). Acc. Chem. Res..

[cit18] Ke W., Stoumpos C. C., Kanatzidis M. G. (2019). Adv. Mater..

[cit19] Even J., Pedesseau L., Jancu J. M. (2015). et al.. J. Phys. Chem. C.

[cit20] Dolomanov O. V., Bourhis L. J., Gildea R. J., Howard J. A. K., Puschmann H. (2009). J. Appl. Crystallogr..

[cit21] Sheldrick G. M. (2015). Acta Crystallogr., Sect. A.

[cit22] Sheldrick G. M. (2015). Acta Crystallogr., Sect. C: Struct. Chem..

[cit23] Brandenburg K. and Putz H., Diamond Crystal Analysis Software, Crystal Impact GbR, Bonn, Germany, 1999

[cit24] Checinska L., Grabowsky S., Malecka M., Rybarczyk-Pirek A. J., Józwiak A., Paulmann C., Luger P. (2011). Acta Crystallogr., Sect. B: Struct. Sci..

[cit25] Seth S. K., Sarkar D., Jana A. D., Kar T. (2011). Cryst. Growth.

[cit26] Seth S. K., Sarkar D., Kar T. (2011). CrystEngComm.

[cit27] Manna P., Seth S. K., Das A., Hemming J., Prendergast R., Helliwell M., Choudhury S. R., Frontera A., Mukhopadhyay S. (2012). Inorg. Chem..

[cit28] Seth S. K., Maity G. C., Kar T. (2011). J. Mol. Struct..

[cit29] Rohl A. L., Moret M., Kaminsky W., Claborn K., McKinnon J. J., Kahr B. (2008). Cryst. Growth.

[cit30] Spackman R., Turner M. J., McKinnon J. J., Wolff S. K., Grimwood D. J., Jayatilaka D., Spackman M. A. (2021). J. Appl. Crystallogr..

[cit31] Spackman M. A., McKinnon J. J. (2002). CrystEngComm.

[cit32] McKinnon J. J., Spackman M. A., Mitchell A. S. (2004). Acta Crystallogr., Sect. B: Struct. Sci..

[cit33] Zhanpeisov N., Matsuoka M., Yamashita H., Anpo M. (1998). J. Phys. Chem. B.

[cit34] : Frisch M. J., Trucks G. W., Schlegel H. B., Scuseria G. E., Robb M. A., Cheeseman J. R., Scalmani G., Barone V., Mennucci B., Petersson G. A., Nakatsuji H., Caricato M., Li X., Hratchian H. P., Izmaylov A. F., Bloino J., Zheng G., Sonnenberg J. L., Hada M., Ehara M., Toyota K., Fukuda R., Hasegawa J., Ishida M., Nakajima T., Honda Y., Kitao O., Nakai H., Vreven T., Montgomery Jr J. A., Peralta J. E., Ogliaro F., Bearpark M., Heyd J. J., Brothers E. N., Kudin K. N., Staroverov V. N., Kobayashi R., Normand J., Raghavachari K., Rendell A., Burant J. C., Iyengar S. S., Tomasi J., Cossi M., Rega N., Millam J. M., Klene M., Adamo C., Cammi R., Ochterski J. W., Martin R. L., Morokuma K., Farkas O., Foresman J. B. and Fox D. J., Gaussian 09, Revision E.01, Gaussian, Inc., Wallingford CT, 2013

[cit35] Nicklass N., Dolg M., Stoll H., Preuss H. (1995). J. Chem. Phys..

[cit36] : Dennington R., Keith T. A. and Millam J. M., GaussView, Version 5, Semichem Inc., Shawnee Mission, KS, 2009

[cit37] Lu T., Chen F. (2012). Multiwfn: A multifunctional wavefunction analyzer. J. Mol. Struct..

[cit38] Tauc J. (1968). Optical properties and electronic structure of amorphous Ge and Si. Mater. Res. Bull..

[cit39] CCDC 2431918: Experimental Crystal Structure Determination, 2025, 10.5517/ccdc.csd.cc2mmlzv

