# Supplementary material for: Structural, optical, thermal, and computational insights into the 1D antimony-based hybrid perovskite (C10H13N4)[SbI4]·H2O
Source: RSC Adv. 2026 May 5;16(26):23335–53. doi: 10.1039/d6ra02020h (PMC13142660; doi:10.1039/d6ra02020h)
Supplement: RA-016-D6RA02020H-s001 [file RA-016-D6RA02020H-s001.pdf]

This journal is © The Royal Society of Chemistry 2026

Supplementary

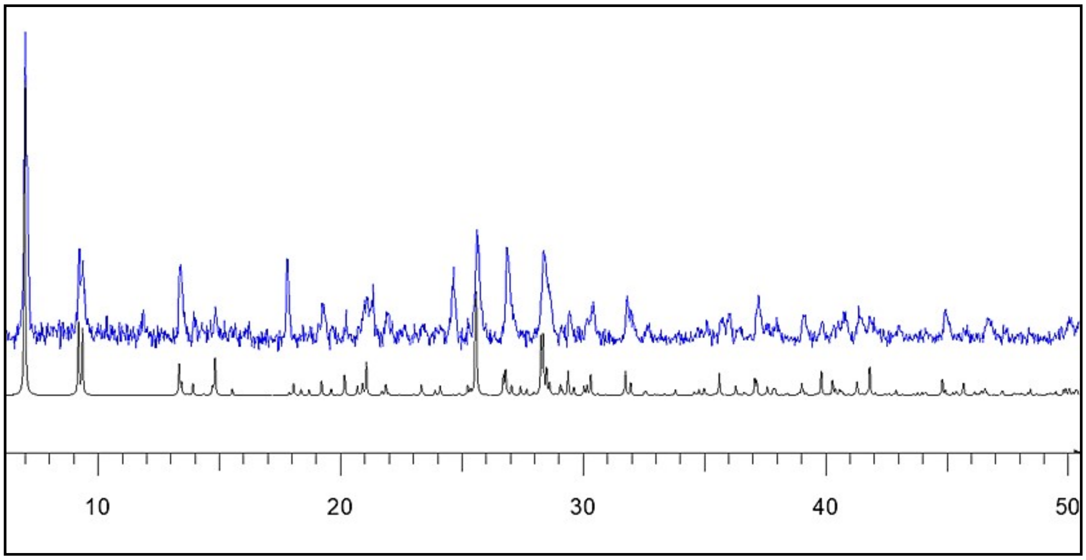

Fig. 1.S: Experimental (blue) vs calculated (black) peaks of PXRD

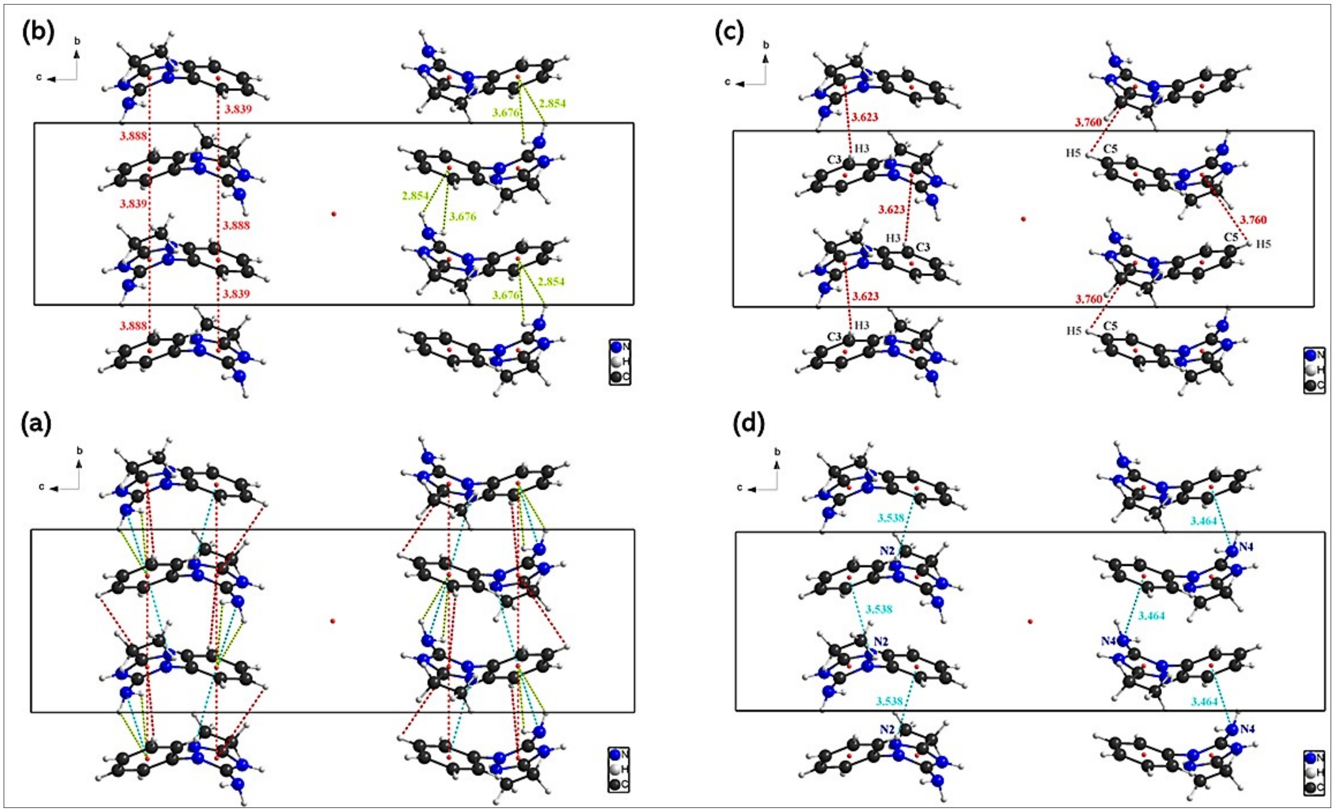

Fig. 2.S: **(a)** all types of π interactions, **(b)** $\pi\cdots\pi$ and N-H$\cdots\pi$ interactions, **(c)** C-H$\cdots\pi$ interactions, **(d)** n$\cdots\pi$ interactions, along the a-axis

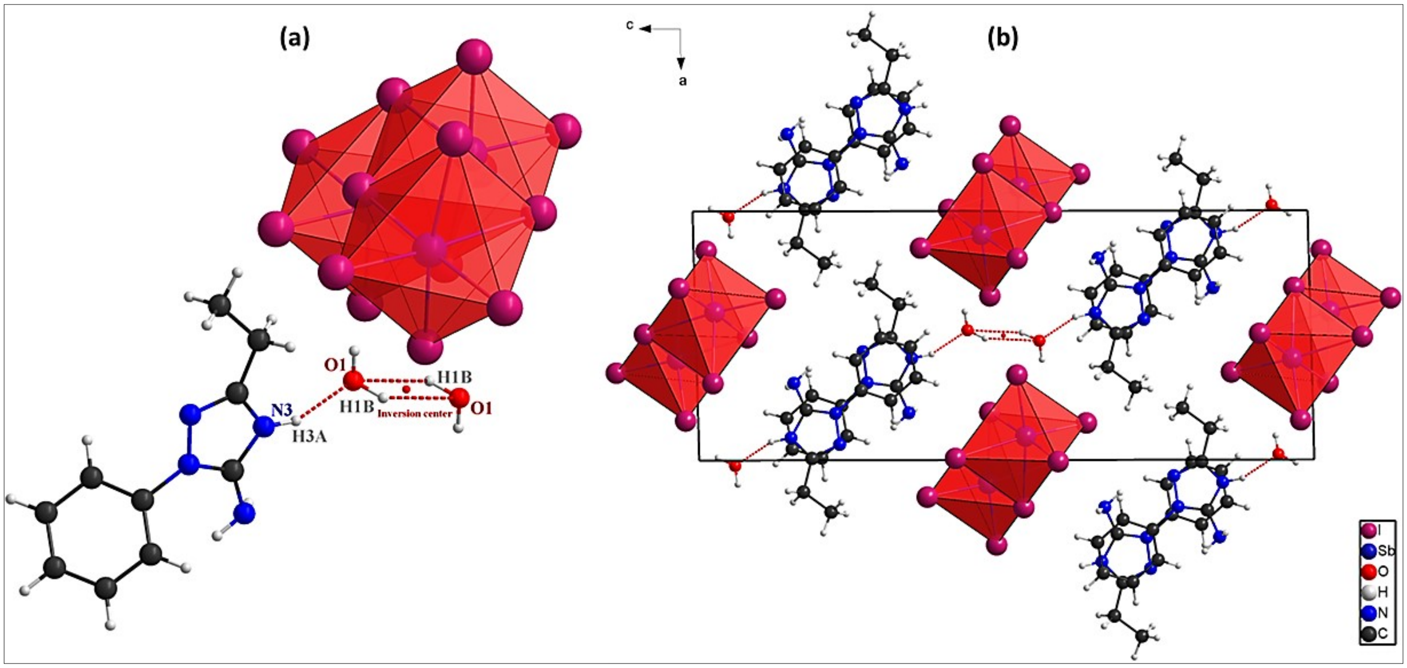

Fig. 3.S: Hydrogen bonding of the titled compound (**(a)** in the formula unit, and **(b)** the unit cell)

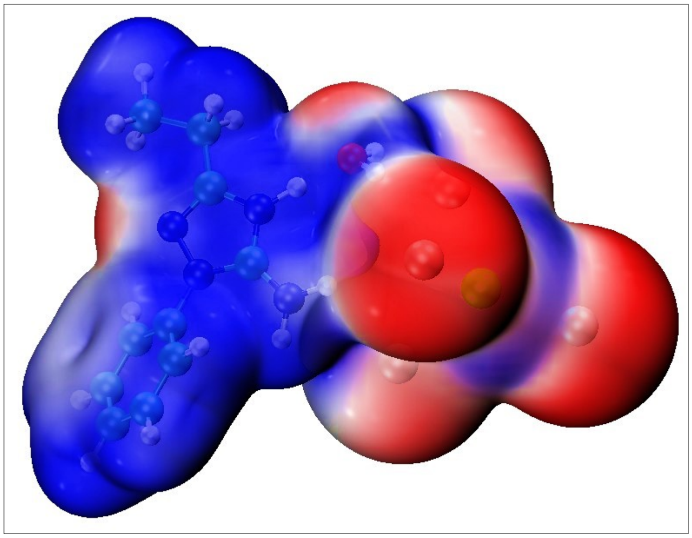

Fig. 4.S: Molecular Electrostatic Potential Analysis of the Target Compound

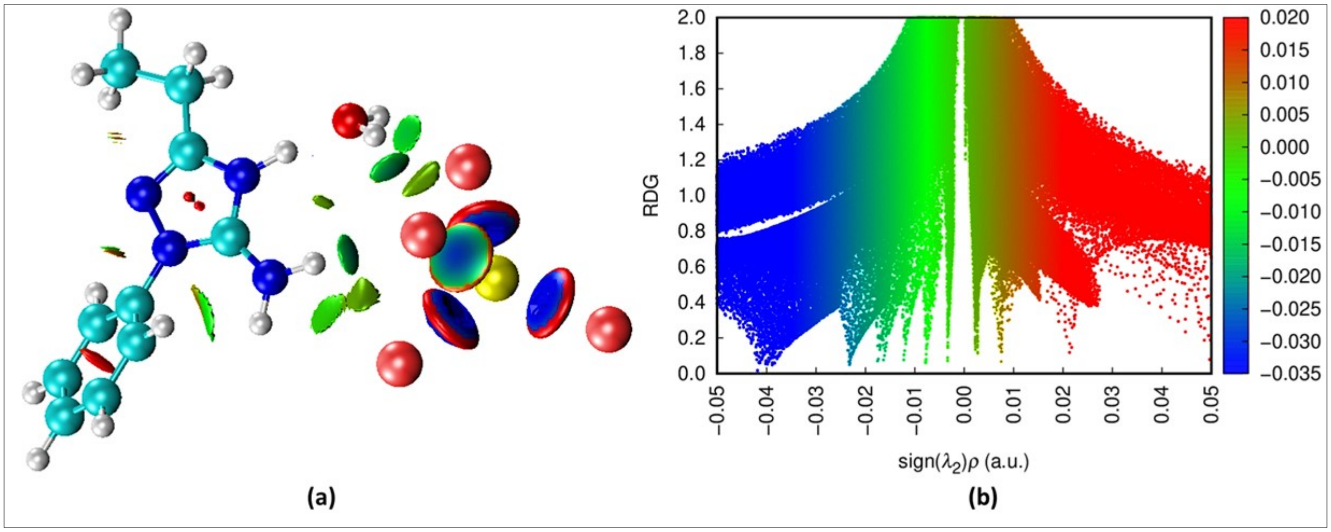

Fig. 5.S: (a) NCI and **(b)** RDG Visualization of the Target Compound

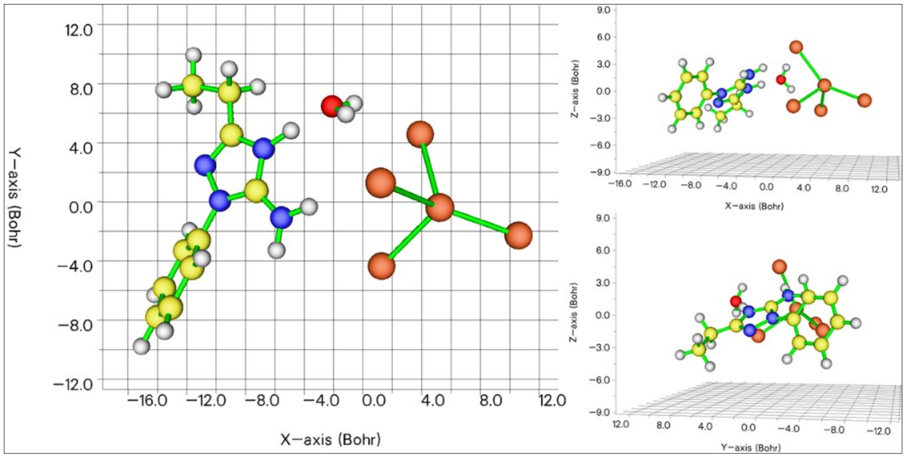

Fig. 6.S: Visualization in (XY), (XZ), and (YZ) planes

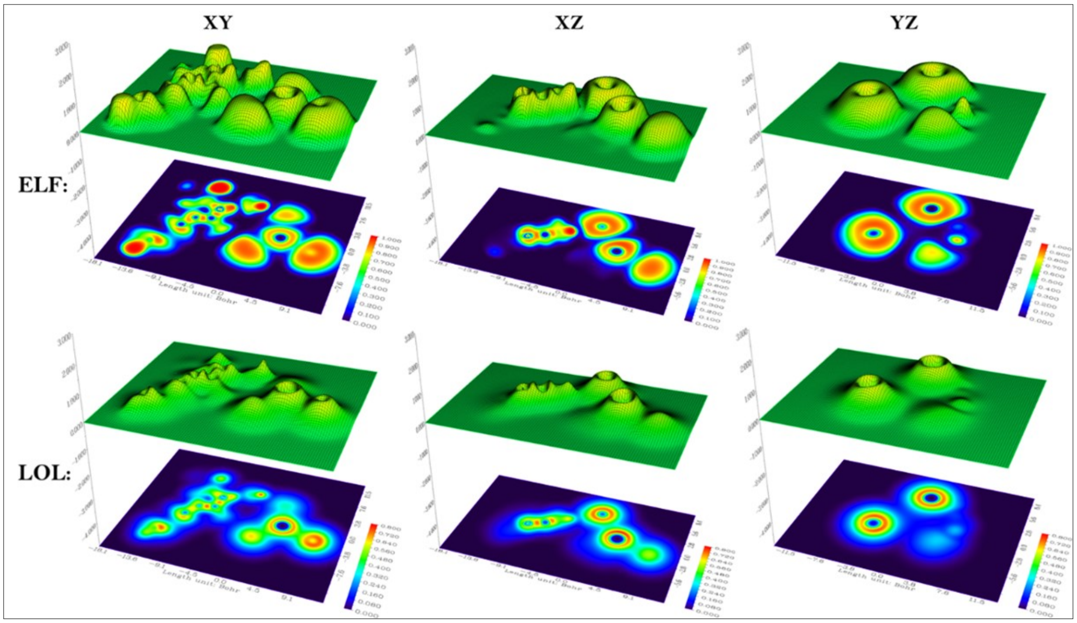

Fig. 7.S: Electron Localization Function (ELF) and LOL analyses

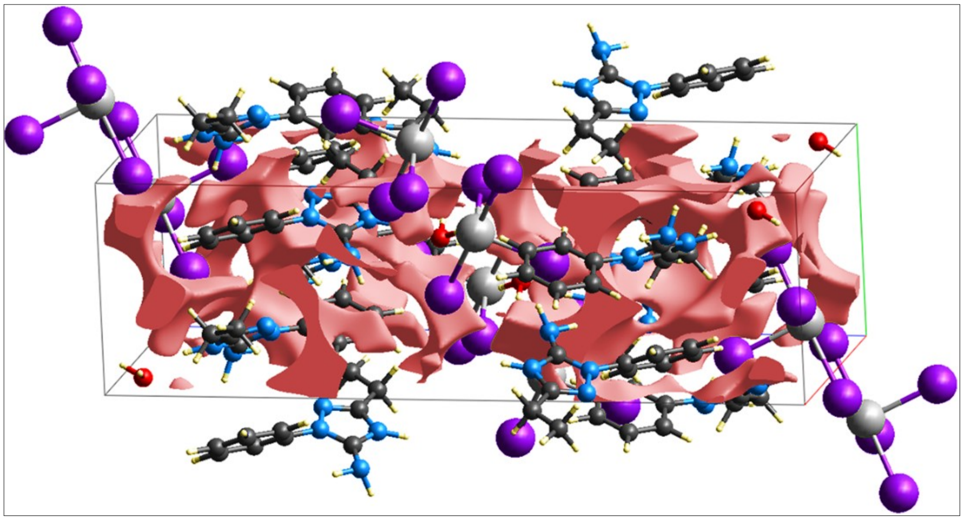

Fig. 8.S: Presence of a Crystal Void in the Compound

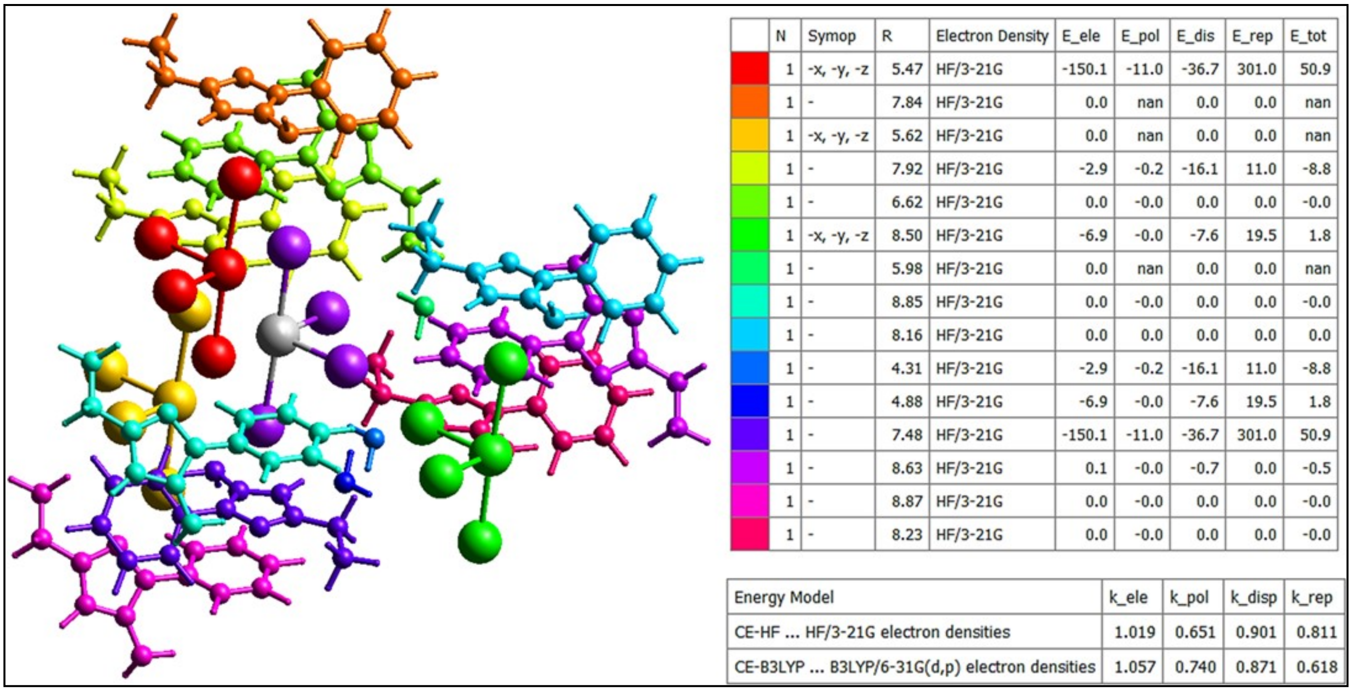

Fig. 9.S: Energy Framework Analysis of Intermolecular Interactions

Table 1.S: The attributions of calculated and observed frequencies of the vibration modes of the compound

IR (cm^{-1})	Raman (cm^{-1})	Calc. wavenumbers (cm^{-1})	Assignement
**3556**	-	**3576**	$\upsilon_{as}(NH_2)$
**3427**	-	**3426**	$\upsilon_{s}(H_2O)$
**3362**	-	**3268**	$\upsilon_{s}(NH_2)$
**3129**	-	**3129**	$\upsilon_{as}(C\text{-}H)$
**3052**	-	**3056**	$\upsilon_{s}(C\text{-}H) + \upsilon_{as}(CH_3)$
**2965**	-	**2966**	$\upsilon_{s}(CH_3) + \upsilon_{as}(CH_2)$
**1800-2800**	-	**2539**	$\upsilon(N\text{-}H....O)$
**1668**	-	**1633**	$\delta(H\text{-}O\text{-}H) + \delta(NH_2)$
**1582**	-	**1565**	$\upsilon(C{=}C) + \delta(CH_2)$
**1491**	-	**1462**	$\omega(CH_3)$
**1453**	-	**1427**	$\gamma(CH_3)$
**1168**	-	**1168**	$\upsilon(C\text{-}C)$
**1017**	-	**1001**	$\omega(NH_2)$
**941**	-	**938**	$\beta(C\text{-}H)$
**849**	-	**870**	$\gamma(C\text{-}H)$
**754**	-	**714**	$\omega(H\text{-}O\text{-}H)$
**668**	-	**634**	$\delta(H\text{-}O\text{-}H)$
**556**	-	**551**	$\rho(C\text{-}C\text{-}C)$
-	-	**293**	$\upsilon_{as}(Sb\text{-}I)$

**-**	**159**	**142**	**υ_s(Sb-I)**
**-**	**108**	**106**	**δ_{as}(I-Sb-I)**
**-**	**86**	**92**	**δ_s(I-Sb-I)**
**-**	**60**	**59**	**Lattice mode**

υ_s :*symmetric stretching*, υ_{as} :*asymmetric stretching*, β : *in plane bending*, γ :*out plane bending*, δ :*scissoring*, ρ :*rocking* ,
ω:*wagging*, τ : Twisting
